# New species and records of Sericini scarab beetles from the Indian subcontinent (Coleoptera, Scarabaeidae)

**DOI:** 10.3897/zookeys.772.25320

**Published:** 2018-07-06

**Authors:** Kolla Sreedevi, Jana Speer, Silvia Fabrizi, Dirk Ahrens

**Affiliations:** 1 Division of Germplasm Collection and Characterization, ICAR- National Bureau of Agricultural Insect Resources P.B. No. 2491, H.A. Farm Post, Bellary Road, Hebbal, Bengaluru-560024; Karnataka, India; 2 Centre for Taxonomy and Evolutionary Research; Zoologisches Forschungsmuseum Alexander Koenig Bonn, Adenauerallee 160, 53113 Bonn, Germany

**Keywords:** chafers, new distribution records, new species, Oriental Region

## Abstract

The current paper presents new locality records, including first state records for Mizoram, of 92 species of Sericini (Coleoptera: Scarabaeidae: Melolonthinae) from the Indian subcontinent. Eight new species are described herein: *Maladera alloservitrita* **sp. n.**, *M. kolasibensis* **sp. n.**, *M. mizoramensis* **sp. n.**, *Neoserica radhanagariensis* **sp. n.**, Serica (s. str.) basantapurensis **sp. n.**, S. (s. str.) mahakaliensis **sp. n.**, S. (s. str.) therathumensis **sp. n.**, and S. (s. str.) zianii **sp. n.**

## Introduction

The taxonomy of the rich fauna of the sericine chafer beetles (Scarabaeidae: Melolonthinae: Sericini) of the Indian subcontinent was revised in detail recently by a series of papers and monographs (see Ahrens 2004, Fabrizi and Ahrens 2014, Ahrens and Fabrizi 2016). In the framework of a collaboration project between German and Indian chafer taxonomists funded by the German Science foundation (DFG), recently, we were able to study further new material from this region, particularly the material preserved in Indian research institutions, and the first results of the examination of these specimens are presented in this paper. Eight new species are discovered and described herein, and new data on the distribution of 84 additional species and one subspecies are given.

## Materials and methods

The principal terminologies and methods used for specimen dissection and genital preparation are described in detail in Ahrens (2004). For identification of the species, genitalia of all male specimens were examined. After examination, male genitalia were glued on a small pointed card. All examined material is cited with the original label contents given in quotation marks, multiple labels are separated by a “/”, additional comments are given in square brackets. Genitalia of the new species were photographed in both lateral and dorsal views using a stereomicroscope Leica M125 with a Leica DFC420C digital camera. The stacking of the separate images was done using Leica Application Suite (V3.3.0) where a number of separate partly focused images were combined in order to obtain an image that was in focus throughout. The resulting images were subsequently digitally edited to remove errors of the Automontage reconstruction and to obtain a white background. New records were commented in case of a new record for a state or country.

### Abbreviations used

**cSZF** collection Stefano Ziani, Forli (Italy);

**NBAIR** National Bureau of Agricultural Insect Resources, Bangalore (India);

**NME** Natural History Museum, Erfurt (Germany);

**NMPC** National Museum of Natural History, Prague (Czech Republic);

**RMNH** Naturalis Biodiversity Centre, Leiden, (Netherlands);

**ZFMK** Zoological Research Museum A. Koenig, Bonn (Germany).

## Systematics

### Maladera alloservitrita sp. n.

Taxon classificationAnimaliaColeopteraScarabaeidae

http://zoobank.org/5F9D0CE2-E368-4F3D-9B64-DE6DBB41CAAD

Figures 1A–E , 5

#### Type material examined.

Holotype. ♂ "India: Kolasib, Mizoram, 24°13'N, 92°40'E, 25.iv.2014, leg. K. Sreedevi/ 919 Sericini: Asia spec./ ICAR-NBAIR-S1" (NBAIR).

#### Description.

Length: 9.5 mm, elytral length: 7.5 mm, width: 6.1 mm. Body oval, uniformly dark brown, dorsal and ventral face dull, head and anterior pronotum moderately shiny, except lateral setae of elytra and pronotum nearly glabrous.

Labroclypeus wide, subtrapezoidal, widest at base and shiny, lateral margins moderately convex and convergent anteriorly, producing an indistinct angle with ocular canthus, not incised before labrum; anterior angles strongly convex; anterior margin weakly sinuate medially; margins weakly reflexed; surface slightly convex, coarsely and densely punctate, with a few single and fine, erect setae on each side behind anterior margin. Frontoclypeal suture finely incised, weakly curved. Smooth area in front of eyes twice as wide as long; ocular canthus short and wide, finely and densely punctate, with a short terminal seta. Frons shiny, in posterior third dull, finely and moderately densely punctate, with a few single and short setae beside eyes. Eyes moderately large, ratio diameter/ interocular width: 0.7. Antenna with ten antennomeres, club with three antennomeres, as long as remaining antennomeres combined. Mentum convexly elevated, anteriorly slightly flattened.

Pronotum widest at base, lateral margins evenly and convexly convergent anteriorly, anterior angles moderately sharp, distinctly produced, posterior angles blunt, moderately rounded at tip; anterior margin with complete marginal line, nearly straight; lateral and lateral anterior margin with long and fine setae; surface finely and densely punctate, with microscopic setae in punctures, otherwise glabrous. Scutellum wide, triangular, punctures fine and moderately dense, glabrous.

Elytra wide, widest at middle, external apical angle strongly rounded, striae finely impressed, finely and densely punctate, intervals weakly convex, finely and moderately densely punctate, with only microscopic setae in punctures; epipleural edge ending at external apical angle of elytra; epipleura with long and sparse setae; apical margin with a broad membranous rim of fine microtrichomes.

Ventral surface coarsely and densely punctate, with microscopic setae in punctures, with a few longer setae on mesosternum and metasternal plate. Mesosternum between mesocoxae as wide as mesofemur. Ratio of length of metepisternum/metacoxa: 1/1.66. Metacoxa glabrous, laterally with a few robust setae. Abdominal sternites finely and moderately densely punctate, with a transverse row of coarse punctures each bearing a robust seta, ultimate sternite with dense and long setae. Pygidium dull, strongly convex, coarsely and moderately densely punctate, with long setae along the apical margin, otherwise with microscopic setae in punctures.

Legs wide; femora with two longitudinal rows of setae. Metafemur shiny, superficially punctate, anterior edge acute, with adjacent serrated line, which is straight and complete, anterior row of setae present but its setae short; posterior ventral margin almost straight, weakly widened in apical half, neither ventrally nor dorsally serrated but smooth, glabrous. Metatibia moderately wide and short, widest at middle, ratio width/length: 1/2.17, sharply carinate dorsally; with two groups of spines, basal one shortly behind middle, apical one at 4/5 of metatibial length, in basal third with a short serrated line parallel to the dorsal margin and 3–4 coarse punctures each bearing a fine seta; lateral face weakly longitudinally convex, impunctate, only basal half with moderately dense and coarse punctures; ventral margin with four equidistant spines; medial face impunctate and glabrous, apex shallowly sinuate interiorly near tarsal articulation. Tarsomeres impunctate dorsally, circular in cross section, with sparse, fine setae ventrally; metatarsomeres ventrally with a strongly serrated carina, subventrally with a second, smooth longitudinal carina; first metatarsomere distinctly shorter than following two tarsomeres combined and slightly longer than dorsal tibial spur. Protibia moderately long, bidentate, teeth moderately large. All claws symmetrical, feebly curved and long, with normally developed basal tooth.

**Figure 1.**
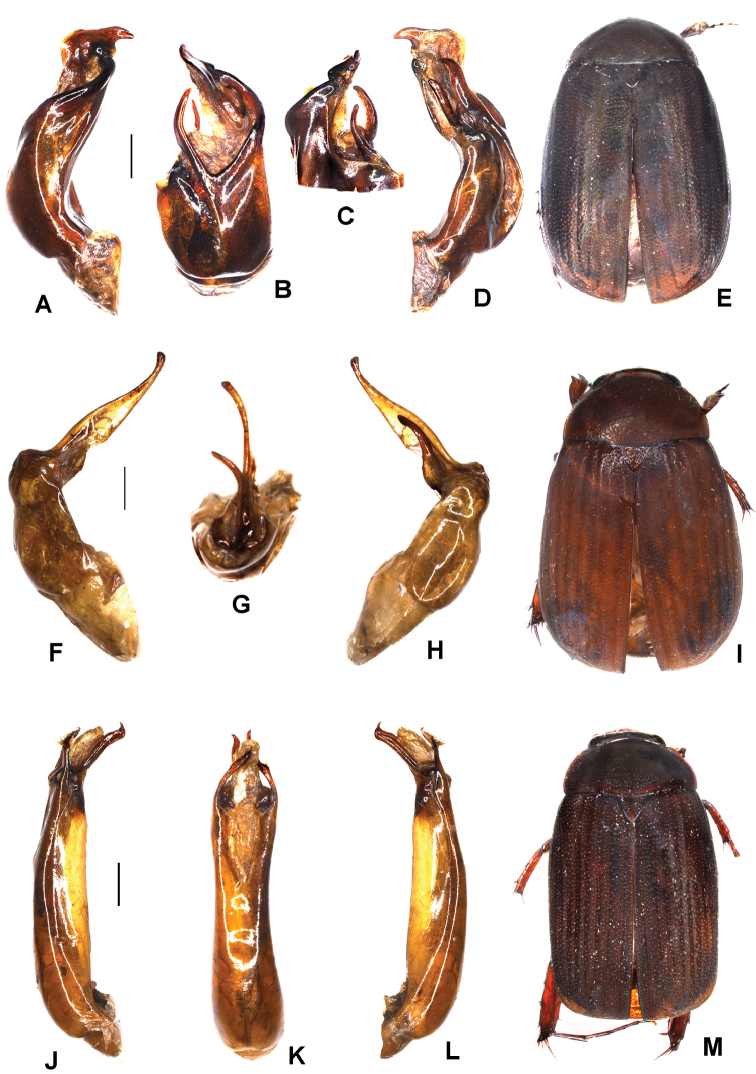
**A–E** *Maladera alloservitrita* sp. n. (holotype) **F–I** *M. kolasibensis* sp. n. (holotype) **J–M** *M. mizoramensis* sp. n. (holotype) **A, F, J** aedeagus, left side lateral view **D, H, L** Aedeagus, right side lateral view **B, G** parameres, dorsal view **K** phallobase, dorsal view. **C** parameres, ventral view **E, I, M** habitus. Scale bar: 0.5 mm. Habitus not to scale.

Aedeagus: Fig. 1A–D. Habitus: Fig. 1E. Female unknown.

#### Diagnosis.

*Maladera alloservitrita* sp. n. resembles *M. servitrita* (Brenske, 1898) in its external and genital morphology. The new species differs from the latter by angles of the dorsomedian process of phallobase and the shape of the parameres: the left paramere is not fused with the phallobase, the right one is short not being extended beyond the apex of the left paramere.

#### Etymology.

The name (adjective in the nominative singular) of the new species is derived from the combined Greek prefix *allo* (other-) and the name *servitrita*, with reference on the high similarity to the closely related species *M. servitrita*.

### Maladera kolasibensis sp. n.

Taxon classificationAnimaliaColeopteraScarabaeidae

http://zoobank.org/ACD98856-8AAA-413D-A322-A00736E28946

Figures 1F–I , 5

#### Type material examined.

Holotype. ♂ "India: Kolasib, Mizoram, 24°13'N, 92°40'E, 25.iv.2014, leg. K. Sreedevi/ 939 Sericini: Asia spec./ ICAR-NBAIR-S2" (NBAIR).

#### Description.

Length: 9.9 mm, length of elytra: 7.5 mm, width: 6.2 mm. Body oval, dark reddish brown, dull, labroclypeus shiny, except some single setae on head dorsal surface nearly glabrous.

Labroclypeus wide and subtrapezoidal, widest at base, lateral margins weakly convex and convergent anteriorly, anterior angles strongly rounded; anterior margin weakly sinuate medially, margins weakly reflexed; lateral margin and ocular canthus produce an indistinct blunt angle; surface nearly flat, finely and densely punctate, glabrous; frontoclypeal suture distinctly incised, evenly curved; smooth area anterior to eye weakly convex, twice as wide as long; ocular canthus short and wide (1/3 of ocular diameter), finely and densely punctate, without a terminal seta. Frons with sparse, fine punctures, and one or two single long setae beside each eye. Eyes small, ratio diameter/interocular width: 0.63. Antenna with ten antennomeres; club with three antennomeres and straight, 1.1 times as long as remaining antennomeres combined. Mentum elevated and slightly flattened anteriorly.

Pronotum moderately wide, widest at base, lateral margins evenly convex and convergent anteriorly, anterior angles distinctly produced and sharp, posterior angles blunt, strongly rounded in the tip; anterior margin convex, with the marginal line widely lacking, base without marginal line; surface sparsely and finely punctate, glabrous; lateral margin finely setose, anterior margin glabrous; hypomeron carinate, not produced ventrally. Scutellum wide, triangular, dull, with fine, dense punctures.

Elytra widest at posterior third, striae distinctly impressed, finely and sparsely punctate, intervals nearly flat, with fine and evenly dense punctures, nearly entirely glabrous; epipleural edge robust, ending at strongly curved external apical angle of elytra, epipleura sparsely setose; apical border of elytra finely membranous, with a fine rim of microtrichomes (visible at ca. 100x magnification).

Ventral surface dull, coarsely and densely punctate, glabrous, metasternal disc sparsely covered with fine, short setae; metacoxa with a few longer setae laterally. Abdominal sternites finely and densely punctate, glabrous, each sternite with a loose transverse row of punctures each bearing a fine seta. Mesosternum between mesocoxae as wide as mesofemur. Ratio of length of metepisternum/metacoxa: 1/1.44. Pygidium weakly convex and dull, coarsely and densely punctate, without midline, glabrous except a few robust setae along apical margin.

Legs short and wide, shiny; femora with two longitudinal rows of setae, finely and sparsely punctate. Metafemur with anterior margin acute, without adjacent serrated line, both rows of setae completely reduced; posterior margin smooth, widened and smooth at apex ventrally, not serrate dorsally, glabrous. Metatibia short and wide, widest at middle, ratio of width/length: 1/2.18, sharply carinate dorsally, with two groups of spines, basal group at anterior third, apical group at three quarters of metatibial length, with a few short robust setae and a short serrated line parallel to the dorsal margin basally; lateral face longitudinally convex, shiny but basal third dull, impunctate and glabrous; ventral margin finely serrate, with four equidistant robust setae; medial face smooth and glabrous; apex finely serrate, moderately truncate interiorly near tarsal articulation. Tarsomeres dorsally smooth and glabrous; meso-and metatarsomeres lacking in holotype. Protibia moderately long, bidentate; anterior claws symmetrical, basal tooth of both claws bluntly truncate at apex.

Aedeagus: Fig. 1F–H. Habitus: Fig. 1I. Female unknown.

#### Diagnosis.

*Maladera kolasibensis* sp. n. is very similar to *M. namborensis* Ahrens & Fabrizi, 2016, in the shape of the genitalia and external morphology. The new species differs from *M. namborensis* by the longer parameres and the lacking lateroapical processes of phallobase.

#### Etymology.

The new species is named after the type locality of the species, Kolasib.

### Maladera mizoramensis sp. n.

Taxon classificationAnimaliaColeopteraScarabaeidae

http://zoobank.org/992D547A-17F0-4957-8DA0-1453153518BB

Figures 1J–M , 5

#### Type material examined.

Holotype. ♂ "India: Kolasib, Mizoram, 24°13'N, 92°40'E, 25.iv.2014, leg. K. Sreedevi/ 940 Sericini: Asia spec./ ICAR-NBAIR-S3" (NBAIR).

#### Description.

Length: 8.9 mm, length of elytra: 5.5 mm, width: 5.1 mm. Body oblong-oval, dorsal face dark brown, ventral face dark reddish brown, dull, head moderately shiny, except some single setae on head dorsal surface nearly glabrous.

Labroclypeus narrow and subtrapezoidal, widest at base, lateral margins straight and convergent anteriorly, anterior angles strongly rounded, anterior margin distinctly sinuate medially, margins moderately reflexed; lateral margin and ocular canthus produce a distinct angle; surface flat, finely and densely punctate, with a few larger punctures each bearing an erect seta; frontoclypeal suture indistinctly incised, evenly curved; smooth area anterior to eye weakly convex, twice as wide as long; ocular canthus short and moderately narrow (1/4 of ocular diameter), finely and densely punctate, terminal seta absent. Frons with dense, fine punctures, with a single long seta beside eyes. Eyes small, ratio diameter/ interocular width: 0.63. Antenna with ten antennomeres; club with three antennomeres and straight, slightly shorter than remaining antennomeres combined. Mentum elevated and slightly flattened anteriorly.

Pronotum transverse, widest shortly before base, lateral margins evenly convex and strongly convergent anteriorly, slightly convexly narrowed towards base; anterior angles distinctly produced and sharp, posterior angles strongly rounded; anterior margin convex, with complete but indistinct marginal line, base without marginal line; surface densely and finely punctate, punctures less dense on midline, with minute setae in punctures; anterior and lateral margin finely setose; hypomeron carinate, not produced ventrally. Scutellum wide, triangular, with fine, dense punctures, punctures less dense on basal midline.

Elytra widest at middle, striae distinctly impressed, finely and sparsely punctate, intervals slightly convex, with fine and dense punctures, with minute setae in punctures, odd intervals with a very few short and white setae; epipleural edge robust, ending at strongly curved external apical angle of elytra, epipleura sparsely setose; apical border of elytra membranous, with a fine rim of microtrichomes (visible at ca. 100 × magnification).

Ventral surface dull, coarsely and densely punctate, glabrous, metasternal disc sparsely covered with fine, short setae; metacoxa with a few longer setae laterally. Abdominal sternites finely and densely punctate, glabrous, each sternite with a transverse row of punctures each bearing a fine seta. Mesosternum between mesocoxae as wide as mesofemur. Ratio of length of metepisternum/metacoxa: 1/1.44. Pygidium moderately convex and dull, coarsely and densely punctate, without impunctate midline, glabrous except a few robust setae along apical margin.

Legs moderately long and wide, shiny; femora with two longitudinal rows of setae, finely and sparsely punctate. Metafemur with anterior margin acute, without adjacent serrated line, anterior row of setae reduced to a few single setae; posterior margin smooth, weakly widened at apex and smooth ventrally, not serrate dorsally, finely shortly setose. Metatibia moderately long and wide, widest at middle, ratio of width/length: 1/2.43, sharply carinate dorsally, with two groups of spines, basal group at middle, apical group at three quarters of metatibial length, with a few robust setae basally subparallel to dorsal margin; lateral face longitudinally convex, shiny, impunctate and glabrous; ventral margin finely serrate, with four equidistant long and robust setae; medial face smooth and glabrous; apex finely serrate, moderately truncate interiorly near tarsal articulation. Tarsomeres dorsally impunctate, glabrous, neither laterally nor dorsally carinate, moderately setose ventrally; metatarsomeres with a strongly serrated ridge ventrally and a smooth subventral longitudinal carina; first metatarsomere as long as following two tarsomeres combined and slightly longer than dorsal tibial spur. Protibia moderately long, bidentate; anterior claws symmetrical, basal tooth of both claws bluntly truncate at apex.

Aedeagus: Fig. 1J–L. Habitus: Fig. 1M. Female unknown.

#### Diagnosis.

*Maladera mizoramensis* sp. n. is in the shape of the genitalia and in its external morphology similar to *M. unguicularis* (Brenske, 1898). The new species differs from *M. unguicularis* by the more elongate phallobase.

#### Etymology.

The name (adjective in the nominative singular) of the new species is derived from its occurrence in Mizoram state of India.

### Neoserica radhanagariensis sp. n.

Taxon classificationAnimaliaColeopteraScarabaeidae

http://zoobank.org/F8C75333-14E7-44D0-9504-F4FF05335043

Figures 2A–D , 5

#### Type material examined.

Holotype. ♂ "India; Radhanagari, Maharashtra 16°22'N, 73°99'E; Anooj & K. Sreedevi 01.vii.2016/ KS0008/ ICAR-NBAIR-S4" (NBAIR). Paratypes: ♂ "India; Radhanagari, Maharashtra 16°22'N, 73°99'E; Anooj & K. Sreedevi 01.vii.2016/ KS0011/ ICAR-NBAIR-S5" (NBAIR), 1 ♂ "India; Radhanagari, Maharashtra 16°22'N, 73°99'E; Anooj & K. Sreedevi 01.vii.2016/ KS0009/ ICAR-NBAIR-S6" (NBAIR), 1 ♂ "India; Radhanagari, Maharashtra 16°22'N, 73°99'E; Anooj & K. Sreedevi 01.vii.2016/ KS0010/ ICAR-NBAIR-S7" (NBAIR), ♂ "India; Radhanagari, Maharashtra 16°22'N, 73°99'E; Anooj & K. Sreedevi 01.vii.2016/ KS0076/ ICAR-NBAIR-S8" (NBAIR).

#### Description.

Length: 7.1 mm, elytral length: 5.5 mm, width: 4.0 mm. Body elongate, yellowish brown, dorsal surface with very dense, simple pilosity and shiny, without dull toment, finely and densely punctate.

Labroclypeus trapezoidal, lateral margins convex and convergent anteriorly, anterior angles moderately convex; anterior margin shallowly sinuate, margins weakly reflexed, lateral margins produce an indistinct angle with ocular canthus; surface weakly convex medially, with fine, very dense punctures, sparsely finely setose; smooth area anterior to eye weakly convex, twice as wide as long; ocular canthus short and wide, finely sparsely punctate, glabrous. Frontoclypeal suture finely incised and moderately curved. Frons with fine and dense punctures, with fine dense but short yellow setae that are bent backwards, without longer setae. Antenna yellow, with ten antennomeres, club in male composed of six antennomeres and straight, 1.2 times longer than remaining antennomeres combined. Eyes moderately large, ratio diameter/interocular width: 0.63. Mentum slightly elevated and convex anteriorly.

Pronotum narrow and elongate; lateral margins in basal half straight and weakly evenly convergent anteriorly, anteriorly margins moderately convex and convergent towards anterior angles; anterior angles sharp and distinctly produced, posterior angles nearly right-angled; base strongly sinuate, without marginal line; surface with fine and shallow, dense punctures, distance between punctures smaller than their diameter, with fine, dense, adpressed setae, on sides with a few long erect setae; anterior and lateral margins with long setae. Scutellum short, triangular, pilosity and punctation similar to that of pronotum.

Elytra elongate, widest in posterior third; striae weakly impressed, finely punctate, intervals flat, finely, evenly and densely punctate, with fine and dense, short setae being directed posteriorly; epipleura with dense and robust setae; apical margin of elytra with fine membranous rim.

Ventral surface including legs finely densely punctate, with dense adpressed setae. Mesosternum between mesocoxae as wide as width of mesofemur. Ratio of length of metepisternum/metacoxa: 1/1.59. Metacoxa with fine and dense adpressed setae on entire surface. Abdominal sternites densely punctate and setose, each sternite with a transversal row of more robust punctures each bearing an erect robust seta. Pygidium distinctly convex, finely and densely punctate, with short fine setae and a few longer erect setae.

Legs moderately long and wide; femora shiny, with two longitudinal rows of setae, finely and densely punctate. Metafemur with anterior margin acute, without adjacent serrated line, anterior row of setae reduced to a few single setae; posterior margin smooth, weakly widened at apex and smooth ventrally, not serrate dorsally, finely shortly setose. Metatibia short, ratio length/with: 1/3.3; dorsal margin carinate, with a fine serrated line beside entire dorsal margin; with two external groups of spines, basal group at half, apical one at three quarter of metatibial length, basally with two single robust setae; lateral face longitudinally convex, finely, evenly and densely punctate, finely setose; ventral margin with robust setae; medial face completely smooth and glabrous, apex near tarsal articulation weakly truncate. Tarsomeres dorsally impunctate and glabrous, sparsely setose ventrally; metatarsomeres ventrally with a strongly serrated carina, subventrally with a second, smooth longitudinal carina, first metatarsomere slightly longer than dorsal tibial spur and as long as following two tarsomeres combined. Protibia moderately long, bidentate. All claws symmetrical, feebly curved and long, with normally developed and simply pointed basal tooth.

**Figure 2.**
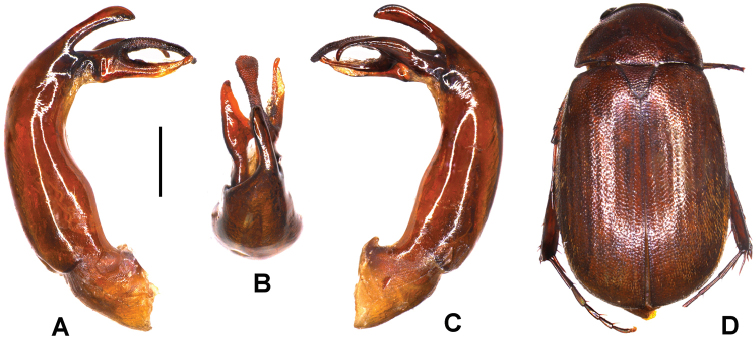
**A–D** *Neoserica radhanagariensis* sp. n. (holotype) **A** aedeagus, left side lateral view **C** aedeagus, right side lateral view **B** parameres, dorsal view **D** habitus. Scale bar: 0.5 mm. Habitus not to scale.

Aedeagus: Fig. 2A–C. Habitus: Fig. 2D.

#### Diagnosis.

This new species is very similar to *Neoserica setigera* (Brenske) in shape of the male genitalia but differs by the shape of right paramere being (in lateral view) much deeper split.

#### Etymology.

The species is name refers to its type locality, Radhanagari; adjective in the nominative singular.

#### Variation.

Length: 6.5-7.1 mm, elytral length: 5.0-5.5 mm, width: 3.9-4.0 mm. Female: Antennal club composed of five antennomeres, 6^th^ antennomere half as long as club; eyes as large as in male; pygidium weakly convex.

### Serica (s. str.) basantapurensis sp. n.

Taxon classificationAnimaliaColeopteraScarabaeidae

http://zoobank.org/903B8236-0031-4E5F-AE30-7734ABA00812

Figures 3A–D , 5

#### Type material examined.

Holotype. ♂ "NEPAL, E, Therathum distr. N Basantapur 2650–2700m, 28/29.V.2016, leg. J. Schmidt 27°10'21"N, 87°25'14"E/ 959 Sericini: Asia spec." (NME).

#### Description.

Length: 8.8 mm, length of elytra: 6.9 mm, width: 4.2 mm. Body oblong, dark brown, antenna yellowish, elytra, legs, and lateral pronotal margins reddish brown, with a few dark impunctate spots on elytra, dorsal surface dull; sparsely setose.

Labroclypeus narrowly subrectangular, slightly wider than long, widest at middle, lateral margins convex and moderately convergent anteriorly; anterior angles weakly rounded, anterior margin deeply and widely sinuate medially; lateral margins weakly reflexed, anterior margin strongly reflexed; surface flat and shiny, finely and densely punctate, without transverse wrinkles, with a few long, erect setae; frontoclypeal suture indistinctly incised, weakly convex; smooth area anterior to eye large and convex, approximately 1.5 times as wide as long; ocular canthus very short and triangular (1/5 of ocular diameter), smooth, with one short terminal seta. Frons completely dull and flat, with fine and moderately dense punctures, with a few erect setae beside eyes and on disc. Eyes large, ratio diameter/ interocular width: 0.83. Antenna yellowish with ten antennomeres; antennomeres three to five nearly as wide as long, antennomere six and seven transverse and short; club with three antennomeres, twice as long as remaining antennomeres combined and weakly reflexed. Mentum weakly elevated, anteriorly flattened. Labrum transverse, short, moderately produced, moderately sinuate medially.

Pronotum transverse, widest at base, lateral margins subparallel and straight, moderately convex and convergent anteriorly; anterior angles weakly produced and rounded, posterior angles moderately rounded; anterior margin strongly convexly and with a robust marginal line; surface moderately densely and finely punctate, a few punctures with white, short and appressed setae; anterior and lateral borders sparsely setose; hypomeron not carinate at base. Scutellum slender and long, triangular, finely and densely punctate, with a few adpressed setae.

Elytra oblong, widest in apical third, striae distinctly impressed, finely and densely punctate, intervals slightly convex, with fine, moderately dense punctures concentrated along striae, intervals with sparsely scattered fine, short, adpressed, white setae; epipleural edge fine, ending at moderately curved external apical angle of elytra; epipleura densely setose, apical border chitinous, without microtrichomes (magnification 100 x).

Ventral surface dull, finely and not densely punctate, moderately densely setose, metacoxa glabrous, with a few long setae only laterally; abdominal sternites finely and densely punctuate, with a transverse row of coarse punctures, each bearing a short seta. Mesosternum between mesocoxae half as wide as mesofemur. Ratio of length of metepisternum/ metacoxa: 1/ 1.32. Pygidium weakly convex and dull, finely and densely punctate, with smooth midline, with sparsely scattered, moderately dense, long setae.

Legs very slender and long; femora with two longitudinal rows of setae, coarsely and not densely punctate between the rows; metafemur shiny, anterior margin acute, without a continuously serrated line behind anterior edge, posterior margin serrated ventrally in apical half and not widened, completely serrated dorsally, in basal half with a few long setae which are half as long as width of metafemur. Metatibia slender and long, widest at apex, ratio of width/ length: 1/ 5.1, dorsally sharply carinate, with two groups of spines, basal group at anterior third, apical group at three quarters of metatibial length, basally with a few single robust setae; external face slightly longitudinally concave, very finely and sparsely punctate, with numerous longitudinal wrinkles; ventral margin serrated, with four robust nearly equidistant setae; medial face flat, glabrous and impunctate, apex interiorly near tarsal articulation distinctly but bluntly truncate. Tarsomeres ventrally with sparse, short setae, dorsally smooth; metatarsomeres laterally and dorsally carinate, with a strongly serrated ridge ventrally; first metatarsomere slightly shorter than following two tarsomeres combined and one third of its length longer than dorsal tibial spur. Protibia long, bidentate, external edge with numerous small teeth, anterior claws asymmetrical, basal tooth of inner claw lobiform and 3/4 as long as apical tooth which is straight.

**Figure 3.**
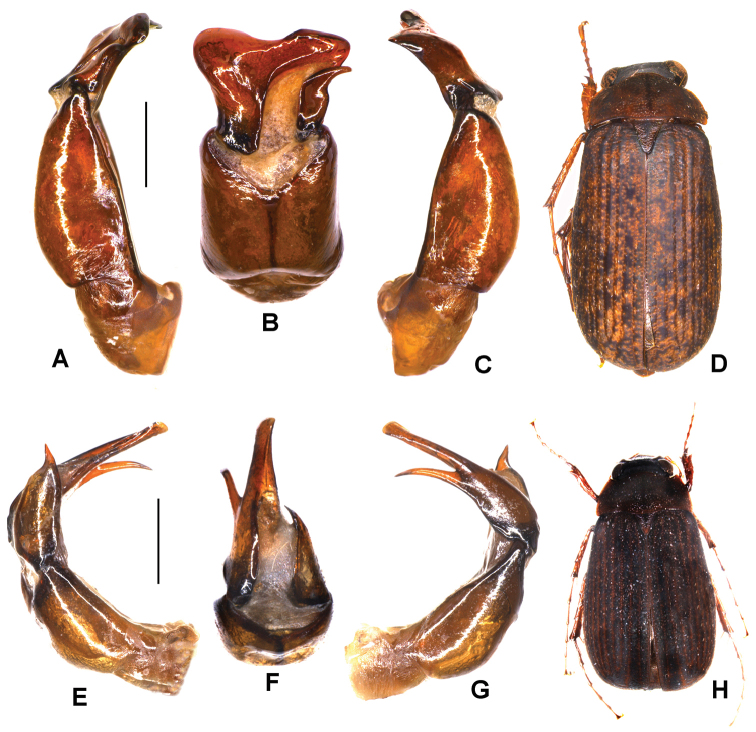
**A–D** *Serica basantapurensis* sp. n. (holotype) **E–H** *S. mahakaliensis* sp. n. (holotype) **A, E** aedeagus, left side lateral view **C, G** aedeagus, right side lateral view **B, F** parameres, dorsal view **D, H** habitus. Scale bar: 0.5 mm. Habitus not to scale.

Aedeagus: Fig. 3A-C. Habitus: Fig. 3D. Female unknown.

#### Diagnosis.

*Serica basantapurensis* sp. n. is in external and genital morphology very similar to *S. bhaktai* Ahrens, 1999 and *S. narya* Ahrens, 1999 from central Nepal and Darjeeling, respectively. The new taxon differs from the two by the apically wide right paramere (distinctly wider than in *S. bhaktai*), whose median apex is not as deeply sinuated as in *S. narya* Ahrens, 1999.

#### Etymology.

The name (adjective in the nominative singular) refers to the type locality close to Basantapur (Nepal).

### Serica (s. str.) mahakaliensis sp. n.

Taxon classificationAnimaliaColeopteraScarabaeidae

http://zoobank.org/A9602DFE-A89C-4C02-BF6B-6261F95DECBB

Figures 3E–H , 5

#### Type material examined.

Holotype: ♂ “NEPAL: Mahakali/Darchula, Bachtal S Thaisain, 2910m, 29°51'52"N, 80°40'17"E, 18.vi.2017, LFF, leg. A. Kopetz #17-08” (NME). Paratypes: 14 ♂, 3 ♀ “NEPAL: Mahakali/Darchula, Bachtal S Thaisain, 2910m, 29°51'52"N, 80°40'17"E, 18.vi.2017, LFF, leg. A. Kopetz #17-08” (NME, ZFMK), 3 ♂ “Mahakali/Darchula, vic. Sintol Montaine Forest, 3050m, 29°49'50.5"N, 80°48'50"E, 26.vi.2017, At light, leg. A. Kopetz #17-14” (NME, ZFMK), 1 ♂ “NEPAL: Mahakali/Darchula, vic. Deuli village, 2400m, 29°48'45"N, 80°47'00"E, 28.vi.2017, LFF, leg. A. Kopetz #17-15” (NME), 2 ♂, 3 ♀ “Mahakali/Darchula, vic. Sitaula, Kopu Lekh, Kulanga Khola, 3500m, 29°53'04"N, 80°44'38"E, 21.vi.2017, river valley, by light, leg. A. Kopetz #17-10” (NME, ZFMK).

#### Description.

Length: 8.1 mm, length of elytra: 5.5 mm, width: 4.6 mm. Body oblong, dark brown, antenna yellowish, ventral surface, elytral striae, legs, and lateral pronotal margins reddish brown, dorsal surface dull; sparsely setose.

Labroclypeus narrowly subtrapezoidal, nearly as wide as long, widest at base, lateral margins straight, strongly convergent anteriorly; anterior angles weakly rounded, anterior margin deeply sinuate medially; lateral margins weakly reflexed, anterior margin strongly reflexed; surface flat and shiny, finely and densely punctate, with a few fine transverse wrinkles and a few erect setae; frontoclypeal suture indistinctly incised, weakly convex; smooth area anterior to eye large and convex, approximately 1.2 times as wide as long; ocular canthus short and triangular (1/4 of ocular diameter), smooth, with one short terminal seta. Frons completely dull and flat, with fine sparse punctures, with a few erect setae beside eyes and on disc. Eyes moderately large, ratio diameter/ interocular width: 0.71. Antenna yellowish with ten antennomeres; antennomeres three to five nearly as wide as long, antennomere six and seven transverse and short; club with three antennomeres, twice as long as remaining antennomeres combined and weakly reflexed. Mentum weakly elevated, anteriorly flattened. Labrum transverse, short, moderately produced, moderately sinuate medially.

Pronotum transverse, widest at base, lateral evenly weakly convex, moderately convergent anteriorly; anterior angles distinctly produced and sharp, posterior angles moderately rounded; anterior margin strongly convexly and with a fine marginal line; surface moderately densely and finely punctate, a few punctures with white, very short and appressed setae; anterior and lateral borders sparsely setose; hypomeron not carinate at base. Scutellum slender and long, triangular, finely and densely punctate, with a few adpressed setae.

Elytra oblong, widest in apical third, striae distinctly impressed, finely and densely punctate, intervals slightly convex, with fine, moderately dense punctures concentrated along striae, intervals with sparsely scattered fine, short, adpressed, white setae; epipleural edge fine, ending at moderately curved external apical angle of elytra; epipleura densely setose, apical border chitinous, without microtrichomes (magnification 100 x).

Ventral surface dull, finely and not densely punctate, moderately densely setose, metacoxa glabrous, with a few long setae only laterally; abdominal sternites finely and densely punctuate, with a transverse row of coarse punctures, each bearing a short seta. Mesosternum between mesocoxae half as wide as mesofemur. Ratio of length of metepisternum/ metacoxa: 1/ 1.37. Pygidium weakly convex and dull, finely and densely punctate, with wide smooth midline, with sparsely scattered, moderately dense, long setae.

Legs very slender and long; femora with two longitudinal rows of setae, coarsely and not densely punctate between the rows; metafemur dull, anterior margin acute, without a continuously serrated line behind anterior edge, posterior margin serrated ventrally in apical half and not widened, completely serrated dorsally, in basal half with a few long setae which are half as long as width of metafemur. Metatibia slender and long, widest at apex, ratio of width/ length: 1/ 5.22, dorsally longitudinally convex, with two groups of spines, basal group at anterior third, apical group at three quarters of metatibial length, basally with a few single robust setae; external face slightly longitudinally concave, very finely and sparsely punctate, without longitudinal wrinkles; ventral margin serrated, with three robust setae of which the apical one is more distant; medial face flat, glabrous and impunctate, apex interiorly near tarsal articulation distinctly but bluntly truncate. Tarsomeres ventrally with sparse, short setae, dorsally smooth; metatarsomeres laterally and dorsally not carinate, with a strongly serrated ridge ventrally; first metatarsomere slightly shorter than following two tarsomeres combined and one third of its length longer than dorsal tibial spur. Protibia long, bidentate, external edge with numerous small teeth, anterior claws symmetrical, basal tooth of inner claw narrow and truncate at apex.

Aedeagus: Fig. 3E–G. Habitus: Fig. 3H.

#### Diagnosis.

*Serica mahakaliensis* sp. n. is in external and genital morphology very similar to *S. kumaonensis* Ahrens, 1999. The new taxon differs by the shorter and wider left paramere, and the lateral elongate branch of the right paramere that inserts at the middle of the paramere in *Serica mahakaliensis* sp. n. (at apical third in *S. kumaonensis*).

#### Etymology.

The name (adjective in the nominative singular) refers to the Mahakali zone (Nepal).

#### Variation.

Length: 8.0-9.5 mm, length of elytra: 5.4-6.5 mm, width: 4.5-5.0 mm. Female: Antennal club slightly shorter than remaining antennomeres combined, eyes small, ratio diameter/ interocular width: 0.5.

### Serica (s. str.) therathumensis sp. n.

Taxon classificationAnimaliaColeopteraScarabaeidae

http://zoobank.org/485AD3BF-B517-4AF4-88C7-637C0158E9FE

Figures 4A–D , 5

#### Type material examined.

Holotype. ♂ "NEPAL, E, Therathum distr. N Basantapur 2650-2700m, 28/29.V.2016, leg. J. Schmidt 27°10'21"N, 87°25'14"E/ 958 Sericini: Asia spec." (NME). Paratypes: 3 ♂ "NEPAL, E, Therathum distr. N Basantapur 2650-2700m, 28/29.V.2016, leg. J. Schmidt 27°10'21"N, 87°25'14"E" (NME).

#### Description.

Length: 7.0 mm, length of elytra: 5.4 mm, width: 3.8 mm. Body oblong, dark brown, antenna yellowish, elytra, legs, and lateral pronotal margins reddish brown, with a few dark impunctate spots on elytra, dorsal surface dull; sparsely setose.

Labroclypeus narrowly subtrapezoidal, wider than long, widest at base, lateral margins moderately convex and convergent anteriorly; anterior angles weakly rounded, anterior margin deeply and widely sinuate medially; lateral margins weakly reflexed, anterior margin strongly reflexed; surface flat and shiny, finely and densely punctate, with shallow transverse wrinkles, with a few long, erect setae; frontoclypeal suture indistinctly incised, weakly convex; smooth area anterior to eye large and convex, approximately 1.5 times as wide as long; ocular canthus very short and triangular (1/4 of ocular diameter), smooth, with one short terminal seta. Frons completely dull and flat, with fine and moderately dense punctures, with a few erect setae beside eyes and on disc. Eyes moderately large, ratio diameter/ interocular width: 0.76. Antenna yellowish with ten antennomeres; antennomeres three to seven transverse and short; club with three antennomeres, three times as long as remaining antennomeres combined and strongly reflexed. Mentum weakly elevated, anteriorly flattened. Labrum transverse, short, moderately produced, moderately sinuate medially.

Pronotum transverse, widest at base, lateral margins nearly straight, moderately convergent anteriorly; anterior angles weakly produced and rounded, posterior angles moderately rounded; anterior margin strongly convexly and with a robust marginal line; surface moderately densely and finely punctate, a few punctures with white, short and appressed setae; anterior and lateral borders sparsely setose; hypomeron not carinate at base. Scutellum slender and long, triangular, finely and densely punctate, with a few adpressed setae.

Elytra oblong, widest in apical third, striae distinctly impressed, finely and densely punctate, intervals slightly convex, with fine, moderately dense punctures concentrated along striae, intervals with sparsely scattered fine, short, adpressed, white setae; epipleural edge fine, ending at moderately curved external apical angle of elytra; epipleura densely setose, apical border chitinous, without microtrichomes (magnification 100 ×).

Ventral surface dull, finely and not densely punctate, moderately densely setose, metacoxa glabrous, with a few long setae only laterally; abdominal sternites finely and densely punctuate, with a transverse row of coarse punctures, each bearing a short seta. Mesosternum between mesocoxae half as wide as mesofemur. Ratio of length of metepisternum/ metacoxa: 1/ 1.31. Pygidium moderately convex and dull, finely and densely punctate, with smooth midline, with sparsely scattered, moderately dense, long setae.

Legs very slender and long; femora with two longitudinal rows of setae, coarsely and not densely punctate between the rows; metafemur shiny, anterior margin acute, without a continuously serrated line behind anterior edge, posterior margin serrated ventrally in apical half and not widened, completely serrated dorsally, in basal half with a few long setae which are half as long as width of metafemur. Metatibia slender and long, widest at apex, ratio of width/ length: 1/ 4.2, dorsally sharply carinate, with two groups of spines, basal group well before middle, apical group at three quarters of metatibial length, basally with a few single robust setae; external face slightly longitudinally concave, very finely and sparsely punctate, with numerous longitudinal wrinkles; ventral margin serrated, with two short but robust widely distant setae; medial face flat, glabrous and impunctate, apex interiorly near tarsal articulation distinctly but bluntly truncate. Tarsomeres ventrally with sparse, short setae, dorsally smooth; metatarsomeres laterally and dorsally carinate, with a strongly serrated ridge ventrally; first metatarsomere slightly shorter than following two tarsomeres combined and one third of its length longer than dorsal tibial spur. Protibia long, bidentate, external edge with numerous small teeth, anterior claws symmetrical, basal tooth of inner claw not lobiform but straight and truncate at apex.

**Figure 4.**
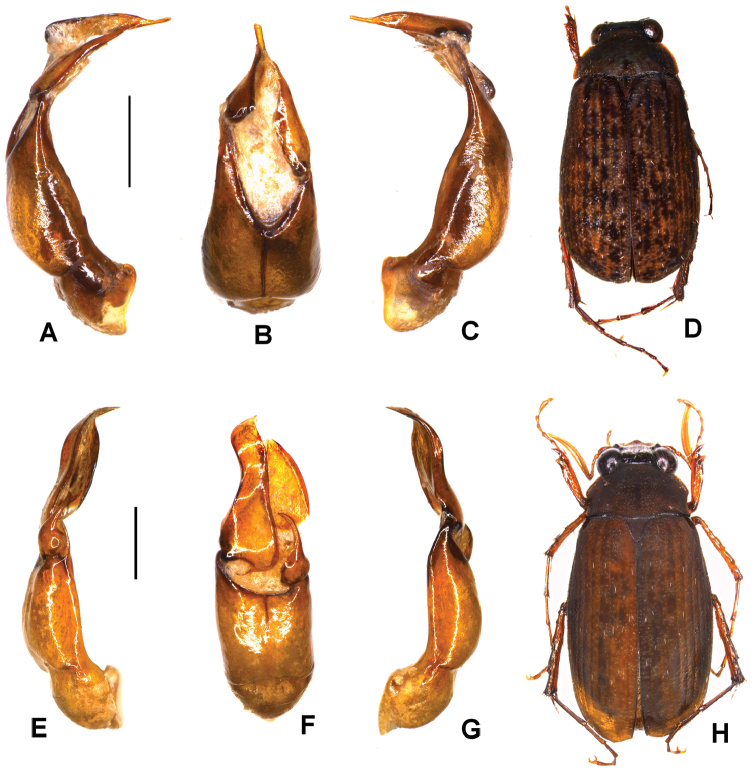
**A–D** *Serica therathumensis* sp. n. (holotype) **E–H** *M. zianii* sp. n. (holotype) **A, E** aedeagus, left side lateral view **C, G** aedeagus, right side lateral view **B, F** parameres, dorsal view **D, H** habitus. Scale bar: 0.5 mm. Habitus not to scale.

Aedeagus: Fig. 4A–C. Habitus: Fig. 4D. Female unknown.

#### Diagnosis.

*Serica therathumensis* sp. n. is very similar to *S. tropdeana* Ahrens, 1999 and *S. thibetana* Brenske, 1897 in external and genital morphology. The new taxon differs from the two by the basal tooth of the inner protarsal claw being straight (not lobiform as in *S. tropdeana* and *S. thibetana*) and truncate at apex as well as the shape of parameres: the left paramere is laterally not widened while the right one is slightly shorter than in *S. thibetana* but distinctly narrower at apical half than in *S. tropdeana*.

#### Etymology.

The name (adjective in the nominative singular) refers to the Therathum district (Nepal).

#### Variation.

Length: 6.5-8.0 mm, length of elytra: 4.9-5.5 mm, width: 3.7-3.8 mm.

### Serica (s. str.) zianii sp. n.

Taxon classificationAnimaliaColeopteraScarabaeidae

http://zoobank.org/A09E027B-2FAC-4D99-9FF5-7B345B33258C

Figures 4E–H , 5

#### Type material examined.

Holotype. ♂ "Bhutan- Wandi Phobjika Valley Tabiting vill. 2900 m, 18.vii.2014, S. Ziani leg." (ZFMK).

#### Description.

Length: 8.0 mm, length of elytra: 5.6 mm, width: 3.8 mm. Body oblong, dark reddish brown, antenna yellowish, legs reddish brown; except head dorsal surface dull, sparsely setose.

Labroclypeus slightly wider than long, widest at middle, lateral margins convex and convergent anteriorly and posteriorly; anterior angles weakly rounded, anterior margin deeply sinuate medially; margins strongly reflexed; surface flat and shiny, finely and densely punctate, with shallow transverse wrinkles on basal part, with numerous long, erect setae; frontoclypeal suture distinctly elevated, weakly curved; smooth area anterior to eye large and convex, approximately 1.5 times as wide as long; ocular canthus short and narrow (1/4 of ocular diameter), smooth, with one short terminal seta. Frons completely shiny and impressed behind frontoclypeal suture, with fine and moderately dense but irregularly scattered punctures, with a few erect setae beside eyes and behind frontoclypeal suture. Eyes very large, ratio diameter/ interocular width: 0.95. Antenna yellowish with ten antennomeres; antennomeres three to seven transverse and short; club with three antennomeres, 1.7 times as long as remaining antennomeres combined and straight. Mentum weakly elevated, anteriorly flattened. Labrum transverse, short, moderately produced, moderately sinuate medially.

Pronotum transverse, widest shortly anterior to middle, lateral margins in basal half deeply concave, convex and convergent in anterior half; anterior angles weakly produced and rounded, posterior angles nearly sharp; anterior margin strongly convexly and with a robust marginal line; surface moderately densely and finely punctate, a few punctures laterally with short and appressed setae, otherwise only with minute setae in punctures; anterior and lateral borders sparsely setose; hypomeron not carinate at base. Scutellum slender and long, triangular, finely and densely punctate, at base impunctate, glabrous.

Elytra oblong, widest at middle, striae distinctly impressed, finely and densely punctate, intervals flat, with fine, moderately dense punctures concentrated along striae, intervals with sparsely scattered fine, short, adpressed, white setae, with a few dark impunctate spots; epipleural edge fine, ending at moderately curved external apical angle of elytra; epipleura densely setose, apical border chitinous, without microtrichomes (magnification 100 ×).

Ventral surface dull, finely and not densely punctate, moderately densely setose, metacoxa glabrous, with a few long setae only laterally; abdominal sternites finely and densely punctuate, with a transverse row of coarse punctures, each bearing a short seta. Mesosternum between mesocoxae half as wide as mesofemur. Ratio of length of metepisternum/ metacoxa: 1/ 1.44. Pygidium moderately convex and dull, finely and densely punctate, with smooth darker midline, with sparsely scattered, moderately dense, long setae.

Legs very slender and long; femora with two longitudinal rows of setae, coarsely and not densely punctate between the rows; metafemur shiny, anterior margin acute, without a continuously serrated line behind anterior edge, posterior margin serrated ventrally in apical half and not widened, completely serrated dorsally, in basal half with a few long setae which are half as long as width of metafemur. Metatibia slender and long, widest at apex, ratio of width/ length: 1/ 5.2, dorsally carinate, with two groups of spines, basal group well before middle, apical group at three quarters of metatibial length, basally with a few single robust setae; external face slightly longitudinally concave, nearly impunctate, with numerous longitudinal wrinkles; ventral margin serrated, with four robust equidistant setae; medial face flat, glabrous and impunctate, apex interiorly near tarsal articulation distinctly but bluntly truncate. Tarsomeres ventrally with sparse, short setae, dorsally smooth; metatarsomeres laterally and dorsally carinate, with a strongly serrated ridge ventrally; first metatarsomere distinctly longer than following two tarsomeres combined and twice as long as dorsal tibial spur. Protibia long, bidentate, external edge basally smooth, anterior claws asymmetrical, basal tooth of inner claw small and lobiform.

Aedeagus: Fig. 4E–G. Habitus: Fig. 4H. Female unknown.

#### Diagnosis.

*Serica zianii* sp. n. is rather similar to *S. olivacea* Brenske, 1896 in external appearance and genital morphology. The new taxon differs by the strongly concave lateral margin of pronotum (in which it is similar to *Serica arborea* Ahrens, 1999 and *S. sticta* Ahrens & Fabrizi, 2009), the larger size, as well as the slightly longer antennal club of male. Genitalia are well distinct from those of *S. olivacea* Brenske, 1896: in *S. zianii* sp. n. the right paramere is only half the length of the left paramere.

#### Etymology.

The name (adjective in the nominative singular) is named after its collector, Stefano Ziani (Forli, Italy).

**Figure 5.**
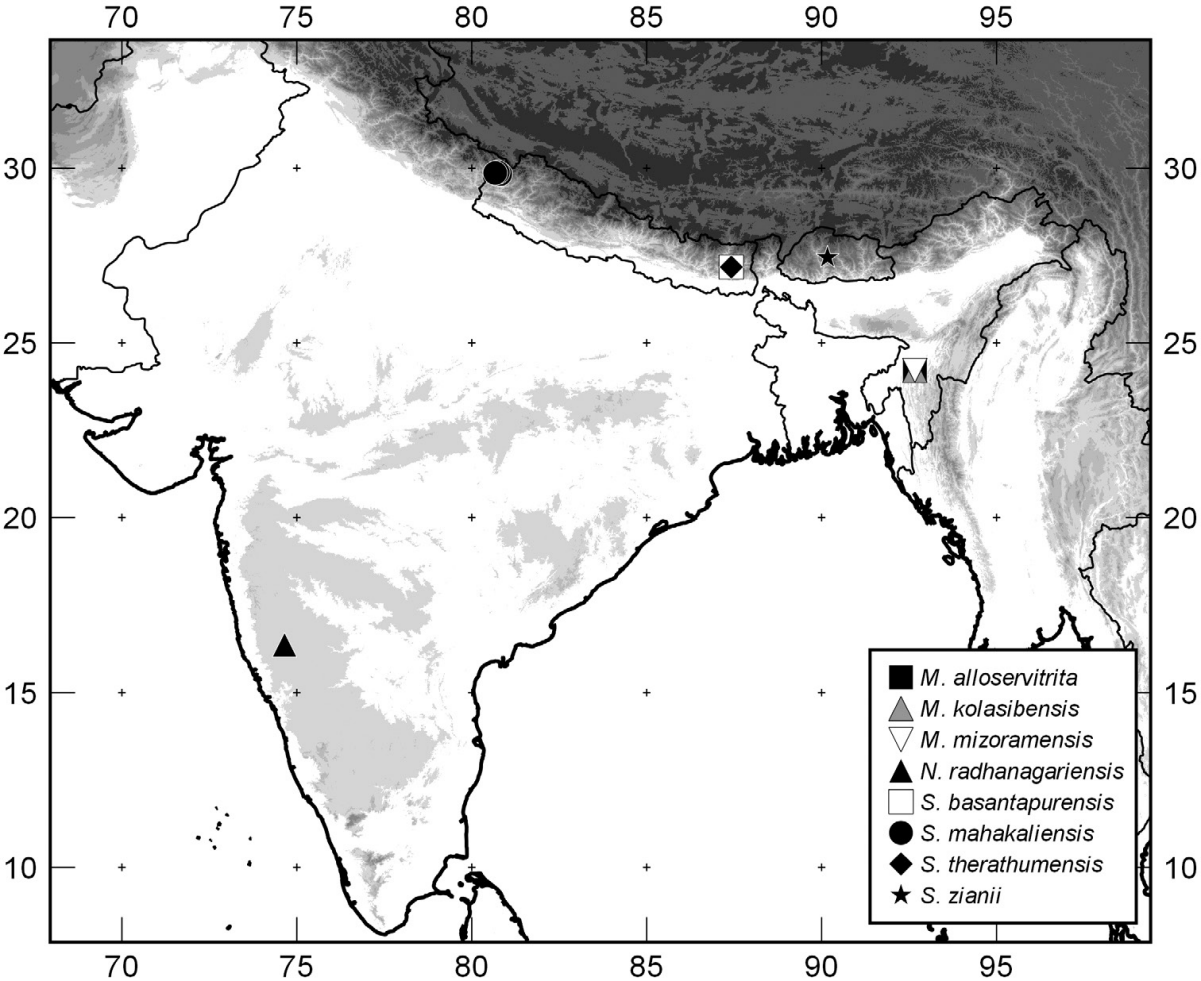
Distributions of the new taxa described in this paper.

### New records

***Amiserica krausei* Ahrens, 2004**

**Material examined.** 1 ♂ “NEPAL: Mahakali/Darchula, Bachtal S Thaisain, 2910m, 29°51'52"N, 80°40'17"E, 18.vi.2017, LFF, leg. A. Kopetz #17-08” (NME), 1 ♀ “NEPAL: Mahakali/Darchula, vic. Deuli village, 2400m, 29°48'45"N, 80°47'00"E, 27-29.vi.2017, at light, leg. A. Kopetz #17-15” (NME), 2 ♀ “Mahakali/Darchula, Godhani, Godhani Khola, 1920 m, 29°49'53"N, 80°40'45"E, 16.vi.2017, at light, leg. A. Kopetz #17-07” (NME).

***Chrysoserica auricoma* (Brenske, 1896)**

**Material examined.** 1 ♂ “India: Meghalaya, Umiam, Barapani, 996 m, 25°68'N, 91°93'E, 3.v.2015, leg. K. Sreedevi” (NBAIR), 1 ♂ “India: Meghalaya, Umiam, Barapani, 996 m, 25°68'N, 91°93'E, 10.v.2016, leg. K. Sreedevi” (NBAIR).

***Chrysoserica stebnickae* Ahrens, 2000**

**Material examined.** 1 ex. “NEP: Narayani/ Makwanpur vic. Naubise, small river vall. Beside street to KTM, 1100m 27°43'16"N, 85°6'56"E, 7.VII.2017, leg. A. Weigel #17-25” (NME).

***Deroserica kulzeri* Frey, 1976**

**Material examined.** 1 ex. “Anaimalai Hills India IV.1978” (RMNH).

***Gynaecoserica marginipes* (Brenske, 1896)**

**Material examined.** 6 ex. “Sikkim Trockenzeit. Fruhstorfer” (RMNH).

***Lasioserica bumthangana* Ahrens, 1999**

**Material examined.** 2 ex. “Bhutan- Bumthang Jakar 2500m 15.vii.2014 S. Ziani leg.” (ZFMK, cSZF).

**Lasioserica maculata ssp. maculata (Brenske, 1894)**

**Material examined.** 1 ex. “Museum Leiden INDIA: U.P.: Bhimtal nr., Naini Tal, 1500 m., 16.VI.1978 F. Smetacek” (RMNH), 1 ex. “Museum Leiden INDIA: U.P.: Bhimtal nr., Naini Tal, 1500 m., 21.VI.1978 F. Smetacek” (RMNH), 2 ex. “Museum Leiden INDIA: U.P.: Bhimtal nr., Naini Tal, 1500 m., 20.VI.1978 F. Smetacek” (RMNH), 1 ex. “Bhimtal N.W. India 25.06.1974 light, 1500 m” (RMNH), 1 ex. “Nepal Seti/ Bajhang 26 km NE Chainpur vic. Shima village/ Ghatganga Khola, 29°43'30"N, 81°21'24"E 2200m, 25.VI.2009 leg. A: Weigel LF #31” (NME), 2 ♂, 2 ♀ “India: Uttarakhand, Ranichauri, 2200 m, 30°32'N, 78°41'E, 19.vi.2017, leg. K. Sreedevi” (NBAIR), 7 ♂, 8 ♀ “NEPAL: Seti/Baihang #35, 19km NE Chainpur, Losani Khola, 2000 m, 29°39'44"N, 81°20'54"E, 27.vi.2009, leg. Weigel” (NME), 15 ♂, 4 ♀ “NEP: Mahakali/Darchula, Godhani, Godhani Khola, 1920 m, 29°49'53"N, 80°40'45"E, 16.vi.2017, at light, leg. A. Kopetz #17-07” (NME), 2 ex. “NEP: Mahakali/Darchula, Godhani, Godhani Khola, 29°49'53"N, 80°40'45"E, 1920 m, 16.VI.2017, by light, leg. A. Weigel #17-07” (NME), 5 ex. “NEP: Mahakali/Darchula, Godhani, Godhani Khola, 29°49'53"N, 80°40'45"E, 1920 m, 17.VI.2017, leg. A. Weigel #17-07” (NME), 1 ♂, 1 ♀ “NEP: Mahakali/Darchula, vic. Panimul, Rugru Gad vall., 2100 - 1800 m, 29°47'58"N, 80°48'36"E, 29.vi.2017, left upp. Slope, rocky meads, open forest, leg. A. Kopetz #17-16” (NME), 2 ♂, 1 ♀ “NEP: Mahakali/Darchula, Godhani to Jatra to Forest SW of Thaisain, 1920 - 2910 m, 29°49'53"N, 80°40'45"E to 29°51'52"N, 80°40'17"E, 18.vi.2017, at light, leg. A. Weigel #17-07a” (NME), 1 ♂ “NEP: Mahakali/Darchula, vic. Sintol Montaine Forest, 3050m, 29°49'50.5"N, 80°48'50"E, 26.vi.2017, At light, leg. A. Kopetz #17-14” (NME), 1 ♂ “Mahakali/Darchula, vic. Deuli village, 2400m, 29°48'45"N, 80°47'00"E, 28-29.vi.2017, LFF, leg. A. Kopetz #17-15” (NME), 2 ex. “NEPAL, Mahakali/Darchula, vic. Dauli village, 2400m, 29°48'45"N, 80°47'0"E, 27-29.VI.2017, leg. A. Weigel KL #17-15” (NME), 1 ex. “NEP: Mahakali/Darchula, vic. Deuli village, 2400m, 29°48'45"N, 80°47'00"E, 28.vi.2017, LFF, leg. A. Kopetz #17-15” (NME), 1 ♂ “NEP: Mahakali/Darchula, vic. Jamir village, Nau Gad Vall., 1380 m, 29°46'59"N, 80°39'18"E, 15.vi.2017, at light, leg. A. Weigel #17-06” (NME), 1 ex. “Nepal, P. Mahakali, D. Darchula, vic. Jamir village, Nau Gad Vall., 15.6.2017 leg. D. Mattern/ 29°46'59"N, 80°39'18"E 1380 m NN” (NME), 2 ex. “Nepal: P: Mahakali D: Darchula, Jamir vill. 15.VI.2017, leg. M. Hartmann, LF, 1380m Nau Gad valley 29.783°N, 80.6540°E 17-#06, tent place” (NME), 2 ex. “Mahakali/Darchula, vic. Sintol Montaine Forest, 3050m, 29°49'50.5"N, 80°48'50"E, 26.vi.2017, At light, leg. A. Kopetz #17-14” (NME).

***Lasioserica nepalensis* Ahrens, 1996**

**Material examined.** 25 ex. “Nepal Seti/ Bajhang 26 km NE Chainpur vic. Shima village/ Ghatganga Khola, 29°43'30"N, 81°21'24"E 2200m, 25.VI.2009 leg. A: Weigel LF #31” (NME), 2 ex. “Nepal Seti/ Bajhang 19 km NE Chainpur Losani Khola, 2000m/ 29°39'44"N, 81°20'54"E 27/28.VI.2009 leg. A. Weigel LF #35” (NME), 24 ♂, 10 ♀ “NEPAL: Mahakali/Darchula, vic. Deuli village, 2400m, 29°48'45"N, 80°47'00"E, 27-29.vi.2017, at light, leg. A. Kopetz #17-15” (NME), 7 ♀ “NEPAL: Mahakali/Darchula, vic. Deuli village, 2400m, 29°48'45"N, 80°47'00"E, 28.VI.2017, LFF, leg. A. Kopetz #17-15” (NME), 1 ♂, 3 ♀ “NEPAL: Seti/Baihang #35, 19km NE Chainpur, Losani Khola, 2000 m, 29°39'44"N, 81°20'54"E, 27.vi.2009, leg. Weigel” (NME), 1 ♂ “Mahakali/Darchula, Godhani, Godhani Khola, 1920 m, 29°49'53"N, 80°40'45"E, 16.vi.2017, at light, leg. A. Kopetz #17-07” (NME), 1 ♂ “Mahakali/Darchula, vic. Lumthi Chamaliva Khola, 1400 m to 1200 m, 29°47'01"N, 80°49'03"E, 16.vi.2017, at light, leg. A. Kopetz #17-17” (NME), 1 ex. “NEP: Mahakali/Dadeldhura vic. Dadeldhura, 3km N of Gaira 29°11'27"N, 80°36'13"E, 2100m 30.VI.2017, leg. A. Weigel #17-18 deciduous forest beside street” (NME).

***Lasioserica nobilis* (Brenske, 1894)**

**Material examined.** 1 ex. “Sikkim Trockenzeit H. Fruhstorfer” (RMNH).

***Lasioserica pilosella* Brenske, 1896**

**Material examined.** 2 ex. “Kurseong N.E. India IV.1957” (RMNH).

***Lasioserica sabatinellii* Ahrens, 1996**

**Material examined.** 1 ex. “NEPAL, E, Therathum distr. N Basantapur 2650-2700m, 28/29.V.2016, leg. J. Schmidt 27°10'21"N, 87°25'14"E” (NME).

***Maladera affinis* (Blanchard, 1850)**

**Material examined.** 2 ex. “Nepal P: Narayani D. Chitwan, Rapti River, Hotel Riverside, 27°34'29"N, 84°29'55"E 160m, 07.VII.2009, leg. M. Hartmann, at light” (NME).

***Maladera alibagensis* Ahrens & Fabrizi, 2016**

**Material examined.** 1 ex. “Museum Leiden SOUTH INDIA 3000 ft Kerala State Trivandrum Dt. Poonmoodi Range V 1972 T.R.S. Nathan” (RMNH).

***Maladera bombycina* Karsch, 1882**

**Material examined.** 1 ♂, 24 ♀ “India: Andhra Pradesh, Tirupati, 153 m, 13°63'N, 79°42'E, 7.iv.2017, leg. K. Sreedevi” (NBAIR).

***Maladera bombycinoides* Ahrens & Fabrizi, 2016**

**Material examined.** 1 ♂ “India: Andhra Pradesh, Bapatla, 7 m, 15°90'N, 80°46'E, 8.vii.2011, leg. Basant, Y. S.” (NBAIR).

**Remarks.** This is the first state record of this species for Andhra Pradesh, being so far known only from the type locality Mysore (Karnataka).

***Maladera breviata* (Brenske, 1898)**

**Material examined.** 1 ex. “M. Lavinia Ceylon VII.1972 light” (RMNH), 2 ♂ “India: Andhra Pradesh, Tirupati, 153 m, 13°63'N, 79°42'E, 28.iv.2017, leg. K. Sreedevi” (NBAIR).

**Remarks.** This is the first state record of this species for Andhra Pradesh, being known only from Sri Lanka and the southern states of India.

***Maladera brevis* (Blanchard, 1850)**

**Material examined.** 1 ♂ “India: Andhra Pradesh, Tirupati, 153 m, 13°63'N, 79°42'E, 7.iv.2017, leg. K. Sreedevi” (NBAIR).

**Remarks.** This is the first state record of this species for Andhra Pradesh, known only from Sri Lanka and the southern states of India.

***Maladera burmeisteri alternans* (Frey, 1975)**

**Material examined.** 1 ♂ “India: Tamil Nadu, Valparai, 1193 m, 10° 32'N, 76° 95'E, 3.vi.2014, leg. Umesh Kumar” (NBAIR), 1 ♂ “Kerala, Wayanad, 770 m, 10°79'N, 76°65'E, 4.v.2015, leg. K. Sreedevi” (NBAIR), 1 ♂ “Kerala, Palakkad, 140 m, 11°69'N, 76°13'E, 12.v.2015, leg. K. Sreedevi” (NBAIR), 1 ♂ “Karnataka, Kodagu, Chettalli, 609 m, 12°33'N, 75°84'E, 6.v.2014, leg. K. Sreedevi” (NBAIR), 1 ♂ “Karnataka, Udupi, 39 m, 13°34'N, 74°74'E, 28.v.2014, leg. Umesh Kumar” (NBAIR).

***Maladera clavata* (Frey, 1972)**

**Material examined.** 1 ♂ “India: Karnataka, Kodagu, Chettalli, 609 m, 12°33'N, 75°84'E, 6.v.2014, leg. K. Sreedevi” (NBAIR).

***Maladera clypeata* (Fairmaire, 1887)**

**Material examined.** 1 ♂ “India: Meghalaya, Umiam, Barapani, 996 m, 25°68'N, 91°93'E, 10.v.2016, leg. K. Sreedevi” (NBAIR), 1 ♂ “India: Mizoram, 888 m, 24°13'N, 92°40'E, 25.iv.2014, leg. K. Sreedevi” (NBAIR).

**Remarks.** This species was recorded first time for the Mizoram state; however, it is known from nearly all neighbouring areas (Ahrens & Fabrizi 2016).

***Maladera dierli* (Frey, 1969)**

**Material examined.** 4 ex. “Museum Leiden INDIA: U.P.: Bhimtal nr., Naini Tal, 1500 m., 9.VI.1978 F. Smetacek” (RMNH), 3 ex. “Museum Leiden INDIA: U.P.: Bhimtal nr., Naini Tal, 1500 m., 10.VI.1978 F. Smetacek” (RMNH), 1 ex. “Museum Leiden INDIA: U.P.: Bhimtal nr., Naini Tal, 1500 m., 6.V.1978 F. Smetacek” (RMNH), 1 ex. “Museum Leiden INDIA: U.P.: Bhimtal nr., Naini Tal, 1500 m., 7.V.1978 F. Smetacek” (RMNH), 3 ex. “Museum Leiden INDIA: U.P.: Bhimtal nr., Naini Tal, 1500 m., 9.VI.1978 F. Smetacek” (RMNH), 6 ex. “Museum Leiden INDIA: U.P.: Bhimtal nr., Naini Tal, 1500 m., 30.VI.1978 F. Smetacek” (RMNH), 10 ex. “Museum Leiden INDIA: U.P.: Bhimtal nr., Naini Tal, 1500 m., 16.VI.1978 F. Smetacek” (RMNH), 2 ex. “Museum Leiden INDIA: U.P.: Bhimtal nr., Naini Tal, 1500 m., 18.VI.1978 F. Smetacek” (RMNH), 2 ex. “Museum Leiden INDIA: U.P.: Bhimtal nr., Naini Tal, 1500 m., 16.VIII.1978 F. Smetacek” (RMNH), 12 ex. “Museum Leiden INDIA: U.P.: Bhimtal nr., Naini Tal, 1500 m., 11.VI.1978 F. Smetacek” (RMNH), 10 ex. “Museum Leiden INDIA: U.P.: Bhimtal nr., Naini Tal, 1500 m., 20.VI.1978 F. Smetacek” (RMNH), 9 ex. “Museum Leiden INDIA: U.P.: Bhimtal nr., Naini Tal, 1500 m., 21.VI.1978 F. Smetacek” (RMNH), 9 ex. “Bhimtal N.W. India 25.6.1974 Light, 1500m” (RMNH), 2 ex. “Museum Leiden INDIA: U.P.: Bhimtal nr., Naini Tal, 1500 m., 31.V.1978 F. Smetacek” (RMNH), 6 ex. “Museum Leiden INDIA: U.P.: Bhimtal nr., Naini Tal, 1500 m., 12.V.1978 F. Smetacek” (RMNH), 8 ex. “Museum Leiden INDIA: U.P.: Bhimtal nr., Naini Tal, 1500 m., 10.VII.1978 F. Smetacek” (RMNH), 2 ex. “Nepal Seti/ Bajhang 26 km NE Chainpur vic. Shima village/ Ghatganga Khola, 29°43'30"N, 81°21'24"E 2200m, 25.VI.2009 leg. A: Weigel LF #31” (NME), 1 ex. “India: Uttarakhand, Ranichauri, 2200 m, 30°32'N, 78°41'E, 19.vi.2017, leg. K. Sreedevi” (NBAIR), 16 ex. “NEPAL: Mahakali/Darchula, Godhani, Godhani Khola, 1920 m, 29°49'53"N, 80°40'45"E, 16.vi.2017, at light, leg. A. Kopetz #17-07” (NME), 1 ex. “NEP: Mahakali/Darchula, Godhani, Godhani Khola, 29°49'53"N, 80°40'45"E, 1920 m, 16.VI.2017, by light, leg. A. Weigel #17-07” (NME), 1 ex. “NEPAL: Mahakali/Darchula, vic. Deuli village, 2400m, 29°48'45"N, 80°47'0"E, 27.VI.2017, LF, leg. A. Weigel #17-15” (NME).

***Maladera emmrichi* Ahrens, 2004**

**Material examined.** 1 ex. “Museum Leiden INDIA: U.P.: Bhimtal nr., Naini Tal, 1500 m., 4.VI.1978 F. Smetacek” (RMNH), 2 ex. “Bhimtal N.W. India 25.6.1974 Light, 1500m” (RMNH), 1 ♂, 3 ♀ “Mahakali/Darchula, Godhani, Godhani Khola, 1920 m, 29°49'53"N, 80°40'45"E, 16.vi.2017, at light, leg. A. Kopetz #17-07” (NME), 1 ex. “NEPAL: Mahakali/Darchula, vic. Deuli village, 2400m, 29°48'45"N, 80°47'0"E, 27.VI.2017, LF, leg. A. Weigel #17-15” (NME), 1 ex. “NEPAL: Seti/Baihang #35, 19km NE Chainpur, Losani Khola, 2000 m, 29°39'44"N, 81°20'54"E, 27.vi.2009, leg. Weigel” (NME).

***Maladera ferekanarana* Ahrens & Fabrizi, 2016**

**Material examined.** 1 ♀ “India: Maharashtra, Radhanagari, 578 m, 16°22'N, 73°99'E, 1.vii.2016, leg. S. Anooj & K. Sreedevi” (NBAIR).

***Maladera ferruginea* (Kollar & Redtenbacher, 1844)**

**Material examined.** 1 ex. “Museum Leiden INDIA: U.P.: Bhimtal nr., Naini Tal, 1500 m., 4.XIII.1978 F. Smetacek” (RMNH), 2 ♂ “India: Himachal Pradesh, Shimla, Kufri, 2630 m, 31°10'N, 77°17'E, 25.vii.2013, leg. K. Sreedevi” (NBAIR), 1 ♂ “India: Himachal Pradesh, Kullu, Nagar, 1279 m, 31°96'N, 77°11'E, 28.vii.2014, leg. K. Sreedevi” (NBAIR), 2 ♂ “NEPAL: Mahakali/Darchula, vic. Deuli village, 2400m, 29°48'45"N, 80°47'00"E, 28.vi.2017, LFF, leg. A. Kopetz #17-15” (NME).

***Maladera fumosa* (Brenske, 1898)**

**Material examined.** 1 ex. “Agumbe Ghat Shimoga Mysore S. India V.1974” (RMNH).

***Maladera garoana* Ahrens & Fabrizi, 2016**

**Material examined.** 1 ♂ “India: Mizoram, Kolasib, 888 m, 24°13'N, 92°40'E, 25.iv.2014, leg. K. Sreedevi” (NBAIR).

**Remarks.** This species is recorded first time from Mizoram state.

***Maladera himalayica himalayica* (Brenske, 1896)**

**Material examined.** 3 ex. “Kurseong N.E. India IV. 1957” (RMNH).

***Maladera himalayica thimphuensis* Ahrens, 2004**

**Material examined.** 2 ex. “Museum Leiden Bhutan: 2300m Lungtenphu 25-VII-1990 Leg. H.R. Feijen” (RMNH), 1 ex. “Museum Leiden Bhutan: Shemgang 30-V-1990 H.R. Feijen” (RMNH), 1 ex. “Bhutan: Thimphu Lungtenphu Alt. 2300m vi-1995 Leg. H.R. Feijen” (RMNH), 2 ex. “Bhutan- Paro Paro 2200m 20.vii.2014 S. Ziani leg.” (cSZF).

***Maladera insanabilis* (Brenske, 1894)**

**Material examined.** 3 ex. “New Delhi India16.7.1977 in spider web” (RMNH), 2 ♂ “India: Uttar Pradesh, Mainpuri, 153 m, 27°23'N, 79°02'E, 5.ix.2007, leg. Satish” (NBAIR), 7 ♂ “India: Himachal Pradesh, Solan, 1325 m, 30°90'N, 77°10'E, 8.vi.2014, leg. K. Sreedevi” (NBAIR), 1 ♂ “India: Delhi, New Delhi, IARI, 216 m, 28°65'N, 77°17'E, 20.vii.2016, leg. K. Sreedevi” (NBAIR), 3 ♂, 6 ♀ “India: Uttar Pradesh, Amroha, Jallopur, 173 m, 28°90'N, 78°46'E, 30.vi.2016, leg. K. Sreedevi” (NBAIR).

***Maladera iridescens* (Blanchard, 1850)**

**Material examined.** 1 ♂, 1 ♀ “India: Haryana, Panchkula, 341 m, 30°69'N, 76°88'E, 11.iv.2016, leg. P. R. Sashank” (NBAIR), 1 ♂ “India: Punjab, Ludhiana 252 m, 30°90'N, 75°86'E, 21.v.2014, leg. K. Sreedevi” (NBAIR).

***Maladera kallarensis* Ahrens & Fabrizi, 2016**

**Material examined.** 1♂ “India: Kerala, Palakkad, 140 m, 11°69'N, 76°13'E, 21.v.2010, leg. Umesh Kumar” (NBAIR).

***Maladera keralensis* (Frey, 1972)**

**Material examined.** 1 ♂ “India: Maharashtra, Radhanagari, 578 m, 16°22'N, 73°99'E, 1.vii.2016, leg. S. Anooj & K. Sreedevi” (NBAIR).

***Maladera lugubris* (Brenske, 1896)**

**Material examined.** 1 ♂ “India: Delhi, New Delhi, IARI, 216 m, 28°65'N, 77°17'E, 6.vi.2014, leg. K. Sreedevi” (NBAIR).

***Maladera luteola* (Moser, 1918)**

**Material examined.** 8 ex. “Jabalpur C. India IX. 1957” (RMNH).

**Remarks.** *Maladera luteola* is known from southern India only; this record from Madhya Pradesh is the northernmost record to date.

***Maladera magnicornis* (Moser, 1920)**

**Material examined.** 1 ♂ “India: Karnataka, Kodagu, Chettalli, 609 m, 12°33'N, 75°84'E, 6.v.2014, leg. K. Sreedevi” (NBAIR).

***Maladera marginella* (Hope, 1841)**

**Material examined.** 1 ♀ “NEPAL: Mahakali/Darchula, Jamir to Godhani, Nau Gad Khola, 29°46'59"N, 80°39'18"E to 29°49'53"N, 80°40'45"E, 1380 - 1920 m, 16.VI.2017, leg. A. Kopetz, #17-06a” (NME).

***Maladera modestula* (Brenske, 1902)**

**Material examined.** 1 ♂, 1 ♀ “Mahakali/Darchula, Godhani, Godhani Khola, 1920 m, 29°49'53"N, 80°40'45"E, 16.vi.2017, at light, leg. A. Kopetz, #17-07” (NME).

***Maladera mollis* (Walker, 1859)**

**Material examined.** 10 ex. “Moragalla Beruwala Sri Lanka XII.1981 light” (RMNH), 3 ex. “Museum Leiden, L.D. Brongersma, Negombo, Ceylon, 21-VIII-1952” (RMNH), 1 ex. “Museum Leiden, L.D. Brongersma, Negombo, Ceylon, 25-27-I-1952” (RMNH), 1 ex. “Mus. Leiden, Negombo, Ceylon, 2.VIII.1956, lgt. V. Akerboom” (RMNH), 1 ex. “Mus. Leiden A. Berger Negombo Ceylon, 22.VIII.1952” (RMNH).

***Maladera paraprabangana* Ahrens & Fabrizi, 2016**

**Material examined.** 1 ♂ “India: Meghalaya, Umiam, Barapani, 996 m, 25°68'N, 91°93'E﻿﻿, 13.v.2016, leg. K. Sreedevi” (NBAIR), 1 ♂ “INDIA: Mizoram, Kolasib, 888 m, 24°13'N, 92°40'E, 25.IV.2014, leg. K. Sreedevi” (NBAIR).

**Remarks.** This species is recorded for first time from Mizoram state; however, it is known from the neighbouring state Meghalaya (Ahrens & Fabrizi 2016).

***Maladera poonmudi* (Frey, 1975)**

**Material examined.** 4 ♂ “India: Maharashtra, Radhanagari, 578 m, 16°22'N, 73°99'E, 1.vii.2016, leg. S. Anooj & K. Sreedevi” (NBAIR), 2 ♂ “India: Tamil Nadu, Valparai, 1193 m, 10°32'N, 76°95'E, 3.vi.2014, leg. Umesh Kumar” (NBAIR), 1 ♂ “Karnataka, Udupi, 39 m, 13°34'N, 74°74'E, 28.v.2014, leg. Umesh Kumar” (NBAIR).

**Remarks.** This species is known from southern India. The new record from Maharashtra is the northernmost occurrence of this species.

***Maladera pseudohongkongica* Ahrens & Fabrizi, 2016**

**Material examined.** 3 ♂ “India: Meghalaya, Umiam, Barapani, 996 m, 25°68'N, 91°93'E﻿﻿, 4.vi.2013, leg. Naveen, M.” (NBAIR).

***Maladera quinquidens* (Brenske, 1896)**

**Material examined.** 1 ex. “Nepal, P: Gandaki, D: Gorkha, Arughat Bazar, Hotel Manaslu 08.V.2013, leg. D. Matern #03/ 28°02'48"N, 84°48'48"E 524 mNN” (NME), 1 ex. “Nep: Mahakali/ Kanchanpur vic. Mahendranagar, Bedkol lake (Siwaliks Mts.), 620m, 29°1'22"N, 80°19'4"E, 02.VII.2017 leg. A. Weigel, KL, #17-20” (NME).

***Maladera rosettae* (Frey, 1972)**

**Material examined.** 9 ex. “Jabalpur C., India, IX.1957” (RMNH).

***Maladera rotundata* (Walker, 1859)**

**Material examined.** 1 ex. “ Candéze, Ceylon” (RMNH).

***Maladera rufocuprea* (Blanchard, 1850)**

**Material examined.** 2 ex. “Jabalpur C. India IX. 1957” (RMNH), 5 ex. “Anamalai Hills, India 5.1977 2700 ft” (RMNH), 2 ex. “Museum Leiden S. INDIA Anamalai Hills Cinchona, 3500 ft V.1968 P. Susai Nathan” (RMNH), 1 ex. “Museum Leiden South India Coimbatore X-1972 T.R.S. Nathan” (RMNH), 2 ex. “Coimbatore S. India 04.1975 “ (RMNH), 1 ex. “Mus. Leiden Negombo Ceylon 4.VIII.1956 I.D. Brangersina” (RMNH), 2 ♂, “India: Karnataka, Belgaum, 784 m, 15°85'N, 74°50'E, 13.ii.2008, leg. Yadav babu” (NBAIR), 1 ♂ “Kerala, Wayanad, 770 m, 10°79'N, 76°65'E, 3.v.2015, leg. K. Sreedevi” (NBAIR), 1 ♂ “Kerala, Palakkad, 140 m, 11°69'N, 76°13'E, 22.v.2010, leg. Umesh Kumar” (NBAIR), 2 ♂, 2 ♀ “India: Maharashtra, Radhanagari, 578 m, 16°22'N, 73°99'E, 1.vii.2016, leg. S. Anooj & K. Sreedevi” (NBAIR).

***Maladera schenklingi* (Moser, 1918)**

**Material examined.** 6 ex. “NEPAL: Narayani/Chitwan, Sauraha, Hotel “Sweet Home”, 180m, 27°35'09"N, 84°29'30.5"E, 04-07.vii.2017, at light, leg. A. Kopetz, #17-22” (NME).

***Maladera sericella* (Brenske, 1898)**

**Material examined.** 1 ♂ “India: Mizoram, Kolasib, 888 m, 24°13'N, 92°40'E, 25.iv.2014, leg. K. Sreedevi” (NBAIR).

**Remarks.** This species is recorded for the first time from Mizoram state; however, it is known from nearly all neighbouring areas (Ahrens & Fabrizi 2016).

***Maladera setosa* (Brenske, 1898)**

**Material examined.** 1 # “NEP: Mahakali/ Kanchanpur vic. Mahadranagar, Shuklaphanta Nature Res., Dsauda river, LFF 28°53'51"N, 80°13'39"E, 160m 1.VII.2017, leg. A. Kopetz, #17-19” (NME).

***Maladera setosiventris* (Moser, 1916)**

**Material examined.** 1 ♂ “India: Kerala, Palakkad, 140 m, 11°69'N, 76°13'E, 22.v.2010, leg. Umesh Kumar” (NBAIR).

***Maladera shiva* Ahrens & Fabrizi, 2016**

**Material examined.** 5 ♂ “India: Mizoram, Kolasib, 888 m, 24°13'N, 92°40'E, 25.iv.2014, leg. K. Sreedevi” (NBAIR).

**Remarks.** This species is recorded for the first time from Mizoram state.

***Maladera simlana* (Brenske, 1898)**

**Material examined.** 13 ex. “Museum Leiden INDIA: U.P.: Bhimtal nr., Naini Tal, 1500 m., 13.VI.1978 F. Smetacek” (RMNH), 11 ex. “Museum Leiden INDIA: U.P.: Bhimtal nr., Naini Tal, 1500 m., 14.VI.1978 F. Smetacek” (RMNH), 2 ex. “Museum Leiden INDIA: U.P.: Bhimtal nr., Naini Tal, 1500 m., 16.VI.1978 F. Smetacek” (RMNH), 2 ex. “Museum Leiden INDIA: U.P.: Bhimtal nr., Naini Tal, 1500 m., 18.VI.1978 F. Smetacek” (RMNH), 1 ex. “Museum Leiden INDIA: U.P.: Bhimtal nr., Naini Tal, 1500 m., 10.VI.1978 F. Smetacek” (RMNH), 8 ex. “Museum Leiden INDIA: U.P.: Bhimtal nr., Naini Tal, 1500 m., 16.VIII.1978 F. Smetacek” (RMNH), 1 ex. “Museum Leiden INDIA: U.P.: Bhimtal nr., Naini Tal, 1500 m., 10.XI.1978 F. Smetacek” (RMNH), 1 ex. “Museum Leiden INDIA: U.P.: Bhimtal nr., Naini Tal, 1500 m., 14.XI.1978 F. Smetacek” (RMNH), 2 ex. “Bhimtal N.W. India 25.6.1974 Light, 1500m” (RMNH), 1 ex. “Nepal Kathmandu Kopundol 1300 Mt. 21-23 V 1976 Leg. W.L. Blom” (RMNH), 1 ♂, 1♀ “India: Uttarakhand, Ranichauri, 2200 m, 30°32'N, 78°41'E, 19.vi.2017, leg. K. Sreedevi” (NBAIR), 2 ex. “NEPAL: Seti/Baihang #35, 19km NE Chainpur, Losani Khola, 2000 m, 29°39'44"N, 81°20'54"E, 27.vi.2009, leg. Weigel” (NME).

***Maladera siniaevi* Ahrens, 2004**

**Material examined.** 1 ♂ “India: Meghalaya, Umiam, Barapani, 996 m, 25°68'N, 91°93'E, 14.v.2016, leg. K. Sreedevi” (NBAIR).

***Maladera siwalikiana* Ahrens, 2004**

**Material examined.** 9 ex. “Nepal P: Narayani D. Chitwan, Rapti River, Hotel Riverside, 27°34'29"N, 84°29'55"E 160m, 07.VII.2009, leg. M. Hartmann, at light” (NME), 1 ♂ “NEPAL: Mahakali/Darchula, Godhani, Godhani Khola, 1920 m, 29°49'53"N, 80°40'45"E, 16.vi.2017, at light, leg. A. Kopetz #17-07” (NME), 1 ♂ “Narayani/Chitwan, Sauraha, Hotel “Sweet Home”, 180m, 27°35'09"N, 84°29'30.5"E, 04-07.vii.2017, at light, leg. A. Kopetz #17-22”(NME), 1 ex. “Narayani/Chitwan, Sauraha, Hotel “Sweet Home”, 27°35'9.7"N, 84°29'29"E, 180m, 04-07.VII.2017, leg. A. Weigel #17-22”(NME), 1 ex. “Narayani/Chitwan, W of Sauraha, small forest with fruit trees, 27°34'48"N, 84°28'10"E, 180m, 05.VII.2017, leg. A. Kopetz #17-22a”(NME).

***Maladera thomsoni* (Brenske, 1894)**

**Material examined.** 14 ex. “Museum Leiden INDIA: U.P.: Bhimtal nr., Naini Tal, 1500 m., 16.VI.1978 F. Smetacek” (RMNH), 5 ex. “Museum Leiden INDIA: U.P.: Bhimtal nr., Naini Tal, 1500 m., 29.V.1978 F. Smetacek” (RMNH), 5 ex. “Museum Leiden INDIA: U.P.: Bhimtal nr., Naini Tal, 1500 m., 4.VI.1978 F. Smetacek” (RMNH), 14 ex. “Museum Leiden INDIA: U.P.: Bhimtal nr., Naini Tal, 1500 m., 7.VI.1978 F. Smetacek” (RMNH), 1 ex. “Museum Leiden INDIA: U.P.: Bhimtal nr., Naini Tal, 1500 m., 8.VI.1978 F. Smetacek” (RMNH), 14 ex. “Museum Leiden INDIA: U.P.: Bhimtal nr., Naini Tal, 1500 m., 9.VI.1978 F. Smetacek” (RMNH), 50 ex. “Museum Leiden INDIA: U.P.: Bhimtal nr., Naini Tal, 1500 m., 10.VI.1978 F. Smetacek” (RMNH), 39 ex. “Museum Leiden INDIA: U.P.: Bhimtal nr., Naini Tal, 1500 m., 11.VI.1978 F. Smetacek” (RMNH), 13 ex. “Museum Leiden INDIA: U.P.: Bhimtal nr., Naini Tal, 1500 m., 12.VI.1978 F. Smetacek” (RMNH), 13 ex. “Museum Leiden INDIA: U.P.: Bhimtal nr., Naini Tal, 1500 m., 14.VI.1978 F. Smetacek” (RMNH), 3 ex. “Museum Leiden INDIA: U.P.: Bhimtal nr., Naini Tal, 1500 m., 17.VI.1978 F. Smetacek” (RMNH), 1 ex. “Museum Leiden INDIA: U.P.: Bhimtal nr., Naini Tal, 1500 m., 1.VII.1978 F. Smetacek” (RMNH), 1 ex. “Museum Leiden INDIA: U.P.: Bhimtal nr., Naini Tal, 1500 m., 21.IX.1978 F. Smetacek” (RMNH), 1 ex. “Museum Leiden INDIA: U.P.: Bhimtal nr., Naini Tal, 1500 m., 12.V.1978 F. Smetacek” (RMNH), 4 ex. “Ghurka valley Nepal 20.4.1976” (RMNH), 8 ex. “Bhimtal N.W. India 25.6.1974 Light, 1500m” (RMNH), 1 ex. “Museum Leiden INDIA: U.P.: Bhimtal nr., Naini Tal, 1500 m., 10.VIII.1978 F. Smetacek” (RMNH), 1 ex. “Museum Leiden INDIA: U.P.: Bhimtal nr., Naini Tal, 1500 m., 16.VIII.1978 F. Smetacek” (RMNH), 1 ♂ “India: Himachal Pradesh, Palampur, 1472 m, 32°11'N, 76°54'E, 18.vi.2014, leg. K. Sreedevi” (NBAIR).

***Maladera trivandrumensis* Ahrens & Fabrizi, 2016**

**Material examined.** 5 ex. “Museum Leiden, SOUTH INDIA: 3000 ft, Kerala State, Trivandrum Dt. Poonmoodi Range, V 1972, T.R.S. Nathan” (RMNH).

***Maladera tumida* Ahrens, 2004**

**Material examined.** 2 ♂ “India: Himachal Pradesh, Solan, Nauni, 1325 m, 30°90'N, 77°10'E, 11.vi.2013, leg. K. Sreedevi” (NBAIR).

***Maladera tyrannica* (Brenske, 1894)**

**Material examined.** 1 ♂ “India: Karnataka, Kodagu, Chettalli, 609 m, 12°33'N, 75°84'E, 8.v.2014, leg. K. Sreedevi” (NBAIR).

***Maladera weigeli* Ahrens, 2004**

**Material examined.** 2 ex. “Nepal Seti/ Bajhang 26 km NE Chainpur vic. Shima village/ Ghatganga Khola, 29°43'30"N, 81°21'24"E 2200m, 25.VI.2009 leg. A: Weigel LF #31” (NME), 1 ex. “Mahakali/Darchula, Gadhani, Godhani Khola, 29°49'53"N, 80°40'45"E, 1920 m, 16.vi.2017, by light, leg. A. Kopetz #17-07” (NME).

***Microserica hispidula* Frey, 1972**

**Material examined.** 1 ex. “Bhutan- Bumthang Thang 3000m Ogyen Choling monoast. 16.vii.2014 S. Ziani leg.” (cSZF), 6 ex. “Bhutan- Bumthang Jakar 2500m 15.vii.2014 S. Ziani leg.” (ZFMK, cSZF).

***Microserica interrogator* (Arrow, 1946)**

**Material examined.** 11 ex. “NEPAL, Bagmati, 16.v.2007, Kathmandu valley, NAGARJUN FOREST RES. Alt. 1400-2100 m. lgt. Fouquét R. +H” (NME), 6 ex. “NEPAL: Mahakali/Darchula, Jamir to Godhani, Nau Gad Khola, 1380 - 1920 m, 29°46'59"N, 80°39'18"E to 29°49'53"N, 80°40'45"E, 16.vi.2017, at light, leg. A. Kopetz, #17-06a” (NME), 1 ex. “Mahakali/Darchula, Godhani, Godhani Khola, 1920 m, 29°49'53"N, 80°40'45"E, 16.vi.2017, at light, leg. A. Kopetz, #17-07” (NME).

***Microserica myagdiana* Ahrens, 1998**

**Material examined.** 1 ex. “Nepal, P: Seti/ D: Baihang way 19 km NE Chainpur (29°39'44"N, 81°20'54"E) to Talkot (29°36'23"N, 81°18'04"E), 2000-1800m, 28.VI.2009 leg. A. Kopetz, #36” (NME), 3 ♂, 1 ♀ “NEPAL: Mahakali/Darchula, Jamir to Godhani, Nau Gad Khola, 1380 - 1920 m, 29°46'59"N, 80°39'18"E to 29°49'53"N, 80°40'45"E, 16.vi.2017, at light, leg. A. Kopetz, #17-06a” (NME).

***Microserica pruinosa* (Hope, 1831)**

**Material examined.** 1 ♂ “Mahakali/Darchula, vic. Panimul, Rugru Gad vall., 2100 - 1800 m, 29°47'58"N, 80°48'36"E, 29.vi.2017, left upp. Slope, rocky meads, open forest, leg. A. Kopetz, #17-16” (NME), 1 ex. “NEPAL: Mahakali/Darchula, vic. Deuli village, 2400m, 29°48'45"N, 80°47'00"E, 27-29.vi.2017, KL, leg. A. Kopetz, #17-15” (NME), 2 ♂ “NEPAL: Mahakali/Darchula, Godhani to Jatra to Forest SW of Thaisain, 1920 - 2910 m, 29°49'53"N, 80°40'45"E to 29°51'52"N, 80°40'17"E, 18.vi.2017, at light, leg. A. Weigel, #17-07a” (NME).

***Microsericaria quadrinotata* (Moser, 1915)**

**Material examined.** 59 ex. “Museum Leiden, SOUTH INDIA, 3000 ft, Kerala State, Trivandrum Dt. Poonmoodi Range, V 1972, T.R.S. Nathan” (RMNH), 10 ex. “Poonmmudi R. Kerala, 1000 m, V.1972 “ (RMNH).

***Neoserica ammattiensis* Ahrens & Fabrizi, 2016**

**Material examined.** 10 ex. “Coimbatore, Madras, India, VI.1973” (RMNH), 3 ex. “Museum Leiden, SOUTH INDIA, Mysore State, 4000 ft, Coorg Distr. Mercara, V.1973 T.R.S. Nathan” (RMNH).

***Neoserica dichroa* Frey, 1973**

**Material examined.** 1 ex. “Museum Leiden, SOUTH INDIA, 300 ft., Kerala State, Trivandrum Dt. Poonmoodi Range, 1972, T.R.S. Nathan”(RMNH).

***Neoserica flavoviridis* (Brenske, 1896)**

**Material examined.** 2 ex. “Anamalai Hills, India, 5.1977, 2700 ft.” (RMNH).

***Neoserica gravida* Ahrens & Fabrizi, 2016**

**Material examined.** 2 ♂ “India: Karnataka, Uttarakannada, 565 m, 14°79'N, 74°69'E, 14.v.2012, leg. Umesh Kumar” (NBAIR).

***Neoserica nathani* Frey, 1972**

**Material examined.** 4 ex. “Poonmoodi R. Kerala V.1972” (RMNH), 2 ex. “Anaimalai Hills, India III 1978” (RMNH), 1 ex. “Museum Leiden, SOUTH INDIA, Mysore State, 4000 ft., Coorg Distr. Mercara, V.1973, T.R.S. Nathan” (RMNH).

***Neoserica periyarensis* Ahrens & Fabrizi, 2016**

**Material examined.** 1 ex. “Museum Leiden, SOUTH INDIA, Coimbatore, X.1972, T.R.S. Nathan” (RMNH).

***Neoserica speciosa* Brenske, 1898**

**Material examined.** 6 ex. “Museum Leiden INDIA: U.P.: Bhimtal nr., Naini Tal, 1500 m., 16.VI.1978 F. Smetacek” (RMNH), 1 ex. “Museum Leiden INDIA: U.P.: Bhimtal nr., Naini Tal, 1500 m., 21.VI.1978 F. Smetacek” (RMNH).

**Remarks.** This species was so faris known only from Meghalaya and the foothills of eastern Himalaya (Assam). The specimens from Uttarakhand state represent a wide disjunction from the currently known range.

***Oxyserica pygidialis annapurnae* (Ahrens, 1995)**

**Material examined.** 8 ex. “Nepal. P. Gandaki; D. Manang way from Yak Kharka to Goa 26.05.2013, leg. D. Mattern #26 / 28°35'50"N, 84°26'51"E 3040mNN to 28°34'00”N, 84°24'13"E 2500m NN” (NME).

***Oxyserica pygidialis pygidialis* Brenske, 1900**

**Material examined.** 1 ♂ “NEPAL: Mahakali/Darchula, Jamir to Godhani, Nau Gad Khola, 1380 - 1920 m, 29°46'59"N, 80°39'18"E to 29°49'53"N, 80°40'45"E, 16.vi.2017, at light, leg. A. Kopetz #17-06a” (NME), 1 # “NEPAL: Mahakali/Darchula, Jamir to Godhani, Nau Gad Khola, 29°46'59"N, 80°39'18"E to 29°49'53"N, 80°40'45"E, 1380 - 1920 m, 16.VI.2017, leg. A. Weigel #17-06a” (NME), 1 ♂ “NEPAL: Mahakali/Darchula, vic. Deuli village, 2400m, 29°48'45"N, 80°47'00"E, 28.vi.2017, LFF, leg. A. Kopetz #17-15”(NME).

***Oxyserica varia* (Frey, 1975)**

**Material examined.** 1 ex. “Bhutan, Thimphu Lungtenphu Alt. 2300m. 13-vii-1996 Leg. H.R. Feijen” (RMNH), 2 ex. “Bhutan, Thimphu Lungtenphu Alt. 2300m. 10-vii-1996 Leg. H.R. Feijen” (RMNH), 24 ex. “Museum Leiden Bhothan: Deepsey 10km SW Thimpho 2200m alt. 5-VI-1990 H.R. Feijen” (RMNH).

***Pachyserica gracilis* Ahrens, 2004**

**Material examined.** 1 ex. “Museum Leiden INDIA: U.P.: Bhimtal nr., Naini Tal, 1500 m., 11.VI.1978 F. Smetacek” (RMNH), 8 ex. “Museum Leiden INDIA: U.P.: Bhimtal nr., Naini Tal, 1500 m., 21.VI.1978 F. Smetacek” (RMNH), 12 ex. “Bhimtal N.W. India 25.6.1974 light, 1500m” (RMNH), 1 ex. “Museum Leiden INDIA: U.P.: Bhimtal nr., Naini Tal, 1500 m., 2.VI.1978 F. Smetacek” (RMNH), 1 ex. “Museum Leiden INDIA: U.P.: Bhimtal nr., Naini Tal, 1500 m., 20.VI.1978 F. Smetacek” (RMNH), 4 ex. “Museum Leiden INDIA: U.P.: Bhimtal nr., Naini Tal, 1500 m., 10.VI.1978 F. Smetacek” (RMNH), 1 ex. “NEPAL, Bagmati, 16.v.2007, Kathmandu valley, NAGARJUN FOREST RES. Alt. 1400-2100 m. lgt. Fouquét R. +H” (NMPC), 2 ex. “Museum Leiden INDIA: U.P.: Bhimtal nr., Naini Tal, 1500 m., 21.VI.1978 F. Smetacek” (RMNH).

***Pachyserica jendeki* Ahrens, 2004**

**Material examined.** 1 ex. “Kurseong N.E. India 04.1957” (RMNH), 1 ♂ “India: Mizoram, Kolasib, 888 m, 24°13'N, 92°40'E, 25.iv.2014, leg. K. Sreedevi” (NBAIR).

**Remarks.** This species is recorded for the first time from Mizoram state; however, it is known from nearly all neighbouring areas (Ahrens & Fabrizi 2016).

***Pachyserica marmorata* (Blanchard, 1850)**

**Material examined.** 2 ex. “Nepal Seti/ Bajhang 26 km NE Chainpur vic. Shima village/ Ghatganga Khola, 29°43'30"N, 81°21'24"E 2200m, 25.VI.2009 leg. A: Weigel LF #31” (NME), 3 ♂, 2 ♀ “NEPAL: Mahakali/Darchula, vic. Deuli village, 2400m, 29°48'45"N, 80°47'00"E, 27-29.vi.2017, at light, leg. A. Kopetz #17-15” (NME), 1 ♀ “NEPAL: Mahakali/Darchula, vic. Deuli village, 2400m, 29°48'45"N, 80°47'00"E, 28.vi.2017, LFF, leg. A. Kopetz #17-15” (NME), 2 ♂, 8 ♀ “Seti/Baihang #35, 19km NE Chainpur, Losani Khola, 2000 m, 29°39'44"N, 81°20'54"E, 27.vi.2009, leg. Weigel” (NME).

**Serica (s. str.) eberti (Frey, 1969)**

**Material examined.** 1 ex. (#) “Nepal Seti/ Bajhang 19 km NE Chainpur Losani Khola, 2000m/ 29°39'44"N, 81°20'54"E 27/28.VI.2009 leg. A. Weigel LF #35” (NME), 4 ♂, 3 ♀ “NEPAL: Mahakali/Darchula, Bachtal S Thaisain, 2910m, 29°51'52"N, 80°40'17"E, 18.vi.2017, LFF, leg. A. Kopetz #17-08” (NME), 1 ex. “NEP: Mahakali/Darchula, Bachtal S Thaisain, 29°51'52"N, 80°40'17"E, 2910m, 18.VI.2017, LFF, leg. A. Weigel #17-08” (NME), 3 ♂, 5 ♀ “Mahakali/Darchula, vic. Sitaula, Kopu Lekh, Kulanga Khola, 3500m, 29°53'04"N, 80°44'38"E, 21.vi.2017, river valley, by light, leg. A. Kopetz #17-10” (NME), 6 ♂, 1 ♀ “Mahakali/Darchula, vic. Sintol Montaine Forest, 3050m, 29°49'50.5"N, 80°48'50"E, 25.vi.2017, At light, leg. A. Kopetz #17-14” (NME), 3 ex. “NEPAL: Mahakali/Darchula, vic. Deuli village, 2400m, 29°48'45"N, 80°47'00"E, 27.-29.vi.2017, at light, leg. A. Kopetz #17-15” (NME).

**Serica (s. str.) khajiaris Mittal, 1988**

**Material examined.** 2 ex. “Nepal Seti/ Bajhang 19 km NE Chainpur Losani Khola, 2000m/ 29°39'44"N, 81°20'54"E 27/28.VI.2009 leg. A. Weigel, LF #35” (NME), 32 ♂ “NEPAL: Seti/Baihang #35, 19 km NE Chainpur, Losani Khola, 2000 m, 29°39'44"N, 81°20'54"E, 27.vi.2009, leg. Weigel” (NME), 2 ♂ “Mahakali/Darchula, vic. Deuli village, 2400m, 29°48'45"N, 80°47'00"E, 28.vi.2017, LFF, leg. A. Kopetz, #17-15” (NME).

**Serica (s. str.) khasiana (Moser, 1918)**

**Material examined.** 2 ex. “Nepal Seti/ Bajhang 26 km NE Chainpur vic. Shima village/ Ghatganga Khola, 29°43'30"N, 81°21'24"E 2200m, 29.VI.2009 leg. A: Weigel LF #31” (NME), 2 ♂, 1 ♀ “NEPAL: Mahakali/Darchula, vic. Deuli village, 2400m, 29°48'45"N, 80°47'00"E, 28.vi.2017, LFF, leg. A. Kopetz #17-15” (NME), 1 ♂ “Mahakali/Darchula, vic. Sintol Montaine Forest, 3050m, 29°49'50.5"N, 80°48'50"E, 26.vi.2017, At light, leg. A. Kopetz, #17-14” (NME).

**Serica (s. str.) mureensis Ahrens, 1999**

**Material examined.** 1 ex. “E-NEPAL Sankhuwasabha/ Therathum Distr., S of Gupha 20-3000m, 26.V.16 lg. Schmidt 27°16'23"N, 87°29'47"E” (NME).

**Serica (s. str.) olivacea Brenske, 1896**

**Material examined.** 1 ex. “Kuseong Darjiling N. India X.1957” (RMNH), 1 ♂ “NEPAL: Seti/Baihang #35, 19km NE Chainpur, Losani Khola, 2000 m, 29°39'44"N, 81°20'54"E, 27.vi.2009, leg. Weigel” (NME).

**Remarks.** *Serica olivacea* is here recorded from Nepal for the first time.

**Serica (s. str.) thibetana Brenske, 1897**

**Material examined.** 8 ♂ “NEPAL: Mahakali/Darchula, Bachtal S Thaisain, 2910m, 29°51'52"N, 80°40'17"E, 18.vi.2017, LFF, leg. A. Kopetz #17-08” (NME), 5 ♂ “Mahakali/Darchula, vic. Sitaula, Kopu Lekh, Kulanga Khola, 3500m, 29°53'04"N, 80°44'38"E, 21.vi.2017, river valley, by light, leg. A. Kopetz #17-10” (NME), 6 ♂ “Mahakali/Darchula, vic. Sintol Montaine Forest, 3050m, 29°49'50.5"N, 80°48'50"E, 25.vi.2017, At light, leg. A. Kopetz #17-14” (NME), 2 ♂ “Mahakali/Darchula, vic. Deuli village, 2400m, 29°48'45"N, 80°47'00"E, 28.vi.2017, LFF, leg. A. Kopetz, #17-15” (NME).

**Serica (s. str.) tongluana Ahrens, 1999**

**Material examined.** 25 ex. “NEPAL, E, Therathum distr. N Basantapur 2650-2700m, 28/29.V.2016, leg. J. Schmidt 27°10'21"N, 87°25'14"E” (NME), 1 ex. “NEPAL, E, Taplejung Dist. W-slope Panthibara 2600-2800m,18/19.V.2016, leg. J. Schmidt 27°24'24"N, 87°45'06"E” (NME).

***Sericania loebli* Ahrens, 2004**

**Material examined.** 1 ex. “Shangla Prov., Swat, Pakistan, 4.08.1979, 2400 m.” (RMNH).

***Xenoserica koshiana* (Ahrens, 1999)**

**Material examined.** 1 ex. “NEPAL, Milke Danda Kha King, 2940 m, 27°20'14"N, 87°29'04"E, S Tamang, 28.V.2010, leg J. Schmidt” (NME).

## Supplementary Material

XML Treatment for Maladera alloservitrita

XML Treatment for Maladera kolasibensis

XML Treatment for Maladera mizoramensis

XML Treatment for Neoserica radhanagariensis

XML Treatment for Serica (s. str.) basantapurensis

XML Treatment for Serica (s. str.) mahakaliensis

XML Treatment for Serica (s. str.) therathumensis

XML Treatment for Serica (s. str.) zianii
